# Aberrant Topologies of Bacterial Membrane Proteins Revealed by High Sensitivity Fluorescence Labelling

**DOI:** 10.1016/j.jmb.2023.168368

**Published:** 2024-01-15

**Authors:** Samuel J. Hickman, Helen L. Miller, Alfredas Bukys, Achillefs N. Kapanidis, Ben C. Berks

**Affiliations:** 1 Department of Biochemistry, University of Oxford, South Parks Road, Oxford OX1 3QU, United Kingdom; 2 Biological Physics Research Group, Department of Physics, University of Oxford, Oxford OX1 3PU, United Kingdom; 3 Kavli Institute for Nanoscience Discovery, University of Oxford, Sherrington Road, Oxford OX1 3QU, United Kingdom

**Keywords:** membrane protein topology, periplasm, fluorescence, Tat protein transport, *Escherichia coli*

## Abstract

The cytoplasmic membrane compartmentalises the bacterial cell into cytoplasm and periplasm. Proteins located in this membrane have a defined topology that is established during their biogenesis. However, the accuracy of this fundamental biosynthetic process is unknown. We developed compartment-specific fluorescence labelling methods with up to single-molecule sensitivity. Application of these methods to the single and multi-spanning membrane proteins of the Tat protein transport system revealed rare topogenesis errors. This methodology also detected low level soluble protein mislocalization from the cytoplasm to the periplasm. This study shows that it is possible to uncover rare errors in protein localization by leveraging the high sensitivity of fluorescence methods.

## Highlights

• Proteins located in cell membranes have a defined orientation that is established during their integration into the membrane.
• Fluorescence-based methods have been developed to detect the topological orientation of proteins in the bacterial cytoplasmic membrane at up to single molecule sensitivity.
• These methods show that a very small proportion of membrane proteins are inserted into the cytoplasmic membrane in the wrong orientation.
• Low level mislocalization of a soluble protein across the cytoplasmic membrane is also detected.
• This study demonstrates the power of fluorescence methods to uncover low frequency events in living cells.

## Graphical abstract

**Figure:**
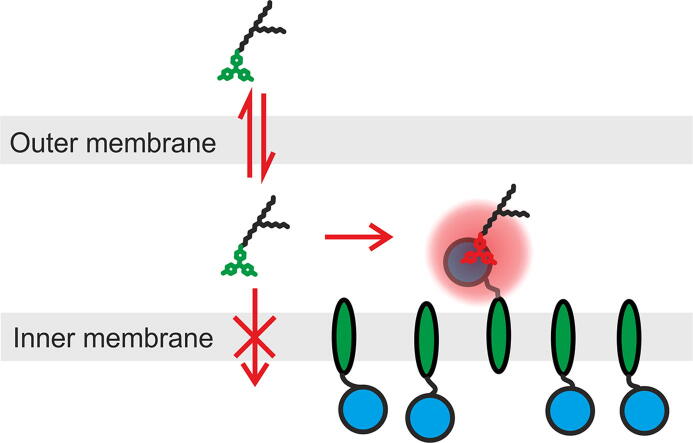

## Introduction

Biogenesis of integral membrane proteins is a complex process that involves the insertion of individual transmembrane helices (TMHs) into the membrane bilayer by either a translocon (protein transporter) or insertase protein[1], 2, 3. It is critical to this process that the TMHs are inserted in the correct orientation (topology) across the membrane. The orientation of the TMHs is ultimately determined by the distribution of basic residues near the helix ends, as encapsulated by the positive-inside rule.^4^ The exact molecular basis by which these residues influence topology is still debated. However, the final orientation of the protein is thought to be established while the protein is associated with the translocon or insertase^5^, except in rare cases where the topology of the protein remains dynamic.^6^ The frequency with which the membrane protein biogenesis apparatus make errors in the topogenesis of native membrane proteins is unknown.

As part of studies of the bacterial Tat protein transport system we developed methodology to determine the topological organisation of membrane proteins in living cells with up to single-molecule sensitivity. This methodology revealed a small proportion of both single and multi-spanning membrane proteins with aberrant topologies, showing that there is a failure rate in membrane protein topology determination. Using this methodology we also discovered that soluble proteins can be mislocalized from the cytoplasm to the periplasm.

### The experimental system

**Figure 1:**
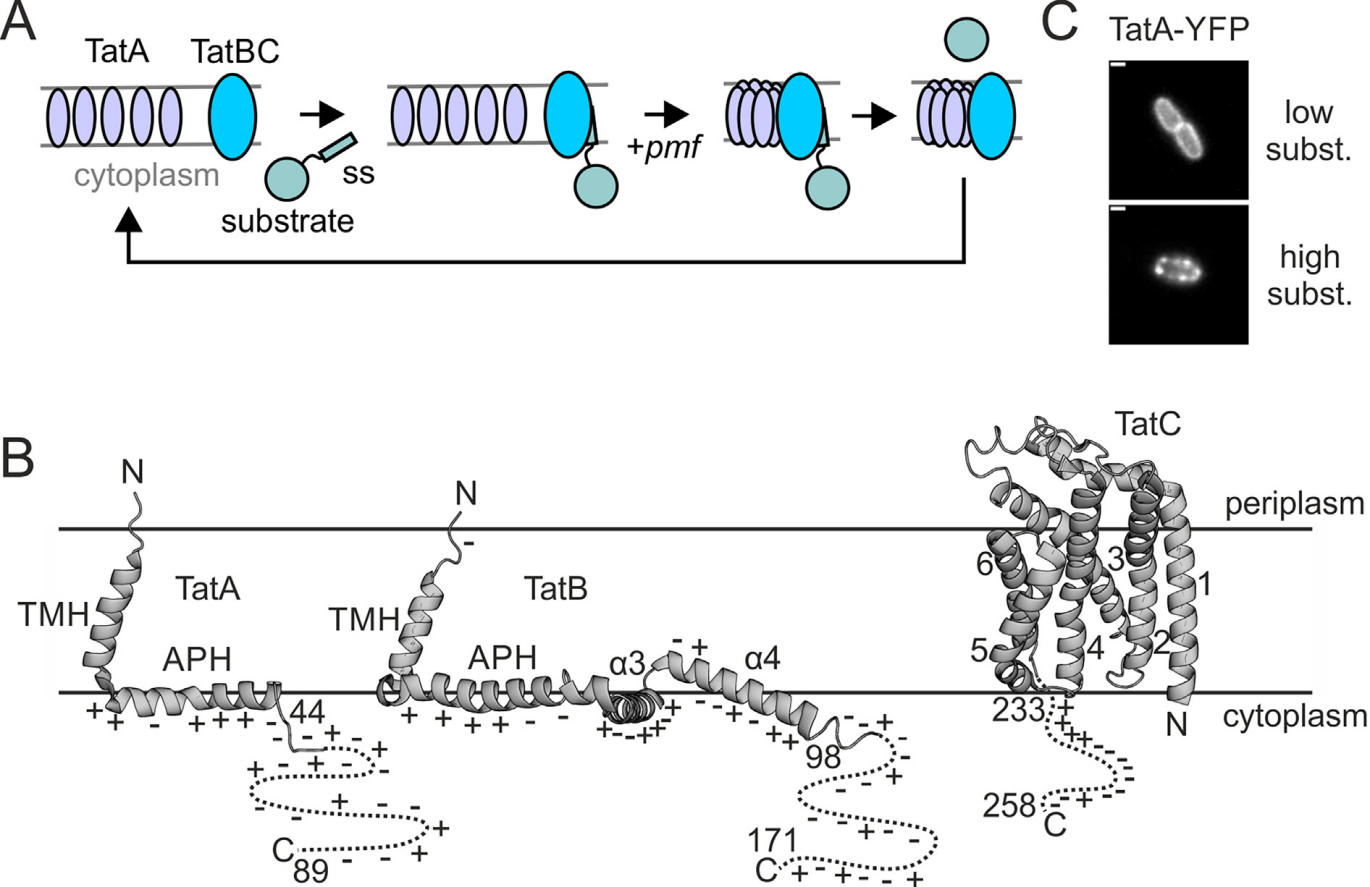
**The proteins of the *E. coli* Tat protein transport system.(**A**)** Schematic model of the Tat transport cycle. Binding of the substate signal sequence (SS) to the TatBC receptor complex triggers a PMF-dependent recruitment and oligomerization of TatA onto TatBC to form the active translocation site. TatA is released from the TatBC complex once substrate transport is complete. **(**B) The normal topological organization of the Tat proteins. The transmembrane (TMH) and amphipathic (APH) helices of TatA and TatB are labelled as well as the third (α3) and fourth (α4) helices of TatB. The TatC TMHs are numbered. The unstructured C-terminal portion of each protein is schematically illustrated by a dashed line bracketed by start and end residue numbers. The distribution of charged residues is indicated for TatA and TatB and for the C-tail of TatC. **(C**) Substrate-induced oligomerization of TatA visualized using a TatA-YFP fusion. TatA-YFP is predominantly in a dispersed state when substrate molecules are scarce (top panel) but predominantly in an oligomerized state when substrates are abundant (bottom panel). Scale bar = 1 µm.

The Tat protein transport system exports folded proteins across the bacterial cytoplasmic membrane.^7^, ^8^ In *Escherichia coli* the Tat system comprises three core membrane proteins TatA, TatB, and TatC. A fourth component, TatE, is structurally and functionally equivalent to TatA but present at much lower levels and is not essential for Tat transport.^9^, ^10^ TatB and TatC form a membrane receptor complex which recognises substrates possessing a Tat-targeting signal peptide.^11^ Binding of a substrate protein to the TatBC complex initiates the recruitment and oligomerization of TatA protomers on to the substrate-TatBC complex in a process that requires the transmembrane proton-motive force (PMF).^12^, ^13^ The assembled TatA oligomer is believed to promote translocation of the substrate protein across the membrane following which TatA disassembles from TatBC. The Tat transport cycle is summarised in Figure 1A.

TatA and TatB are homologous proteins in which a N-terminal TMH is immediately followed by an amphipathic helix (APH) and then a long unstructured tail (Figure 1B).^14^, ^15^ Biochemical studies indicate that TatA and TatB have a N-Out, C-In topology (N terminus in the periplasm, C terminus in the cytoplasm).^16^, ^17^, ^18^ TatC contains six TMHs which adopt a N-In, C-In topology^19^ (Figure 1B).

The substrate-triggered oligomerisation of TatA has been most directly demonstrated by in-cell imaging of fusion constructs in which yellow fluorescent protein (YFP), or another related fluorescent protein (FP), has been fused to the C terminus of TatA (Figure 1C).^12^, ^20^, ^21^, ^22^, ^23^, ^24^ However, these TatA-FP constructs have much reduced transport activity (see e.g. Figure S1D), and in the absence of the native TatA protein or its homologue TatE are unable to support Tat transport at all.^12^, ^22^ The reason the appended FP domain is inhibitory to Tat transport is unknown. It does not prevent TatA oligomerisation or disassembly^12^. Steric occlusion is also unlikely as the FP domain is separated from the APH by the 45 amino acids of the unstructured C-tail, and in most published experiments by an additional 43-amino-acid rigid linker sequence^12^ (although the possibility that the C-tail becomes structured on TatA oligomerization cannot be totally excluded at this point). Instead, we hypothesise that the assembled Tat translocation site attempts to transport the folded FP domain appended to each TatA protomer, and that these transport attempts competitively impede the transport of authentic Tat substrates. If this model is correct, it has important implications for Tat mechanism since it suggests that mere proximity of a folded protein to an active translocation site is sufficient to initiate the transport reaction.

If the FP domains appended to TatA are competing with substrate proteins for transport, then the unstructured C-tail of TatA should be long enough to allow the attached FP to be fully transported across the membrane. Thus, a prediction of the competition model is that the FP domain appended to TatA will be detected in the periplasm. We set out to test this prediction. Since complete transport of the appended domain might be an infrequent occurrence, or might be reversible and thus transient, we sought to maximise the chances of capturing possible translocation events by developing methods to detect the periplasmic exposure of protein domains in living cells with up to single-molecule sensitivity. Using these techniques, we detected low levels of periplasmic exposure for reporter domains appended to not only TatA, but also TatB and TatC. Unexpectedly, these topologies were achieved even with an inactive Tat system and so these observations do not support the competition model. We conclude that the very high sensitivity of our analytical methods allows the detection of rare occurrences of incorrect topogenesis during the process of membrane protein biogenesis. Although developed specifically to test Tat mechanism, our methods will have general utility in assessing protein compartmentalisation in live bacteria.

## Results

### Identification of a periplasm-specific fluorogenic ligand

In order to test the hypothesis that the Tat system transports proteins fused to the C terminus of TatA across the cytoplasmic membrane we developed a high-sensitivity fluorescence imaging method to detect entry of proteins into the periplasm of live *E. coli* cells. This method uses the fluorogen-activating protein dL5 (^25^ where it is referred to as dL5**). dL5 is a covalent dimer of immunoglobin single-chain variable fragments (scFvs) that binds the dye malachite green (MG) to produce fluorescence turn on through a ∼20,000-fold increase in MG fluorescence.^25^ Two MG derivatives that have differential permeability to mammalian cells were investigated for use as compartment-specific probes of dL5 location in *E. coli*. In mammalian cells the dye se-red-s is membrane-permeable^26^ while the dye se-red-xc is membrane-impermeable.^27^

**Figure 2:**
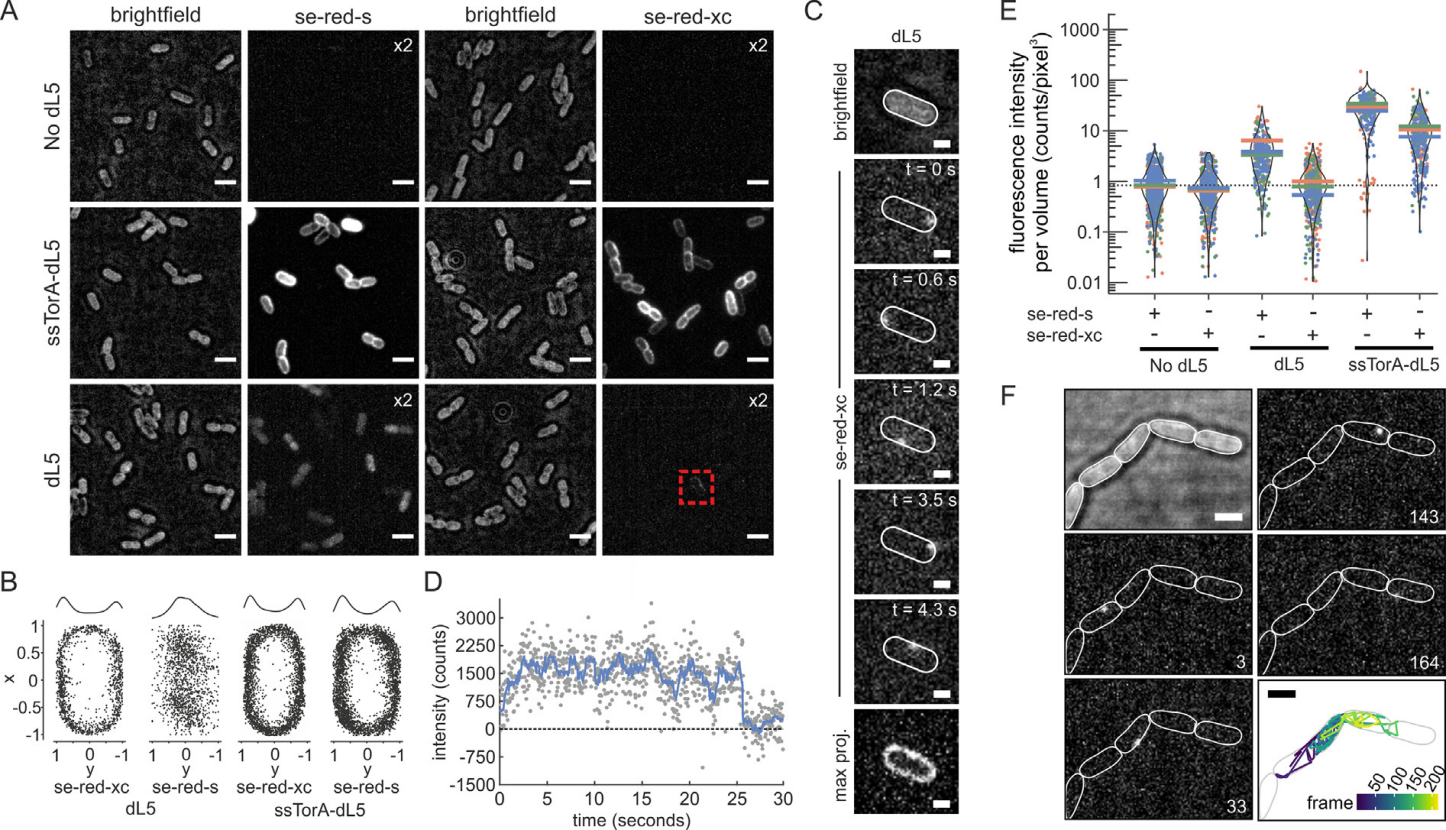
**Selective labelling of periplasm-localized dL5.** Cells expressing low (uninduced) levels of cytoplasmic dL5 or periplasm-targeted ssTorA-dL5 were imaged in the presence of either se-red-s or se-red-xc dyes. (A) Fluorescence images corresponding to 250 frame maximum intensity Z-projections and displayed at standardized brightness except as indicated by multiplication signs. The corresponding brightfield images are to their right. The red box highlights a cell containing a single molecule of labelled dL5 (as determined by photobleaching analysis) with peripheral localization. Scale bars = 3 μm. The image data are representative of cells from at least three independent cultures. (B) Superimpositions of the super-resolution fluorescence localisations from 8 cells for each of the indicated conditions. The data were collected at 30 Hz for 250 frames and normalised by cell dimensions. For dL5 in the presence of se-red-xc, only data from cells exhibiting fluorescence were analysed. The density distributions of the localizations along the short (y) axis of the cell are plotted above the images. (C) Trajectory of a representative single fluorescent dL5 molecule labelled with se-red-xc dye in a cell expressing dL5. The bottom panel shows the maximum intensity Z-projection of 895 frames. Scale bars = 1 μm. (D) Single step photobleaching of the molecule tracked in panel C. The blue line is a Chung-Kennedy filtered^56^ fit with a width of 12 frames (400 ms). The black dotted line is the background baseline. (E) Distribution plots of the fluorescence intensity per cell volume from three biological repeats for each experimental condition for >100 individual cells per repeat. Each colour represents a biological repeat, each point is a single cell measurement, and the coloured horizontal bars represent the mean peak intensity of each biological repeat. The distribution of each full data set is shown by the violin plot. The dotted line is the mean intensity per volume of cells imaged with no dye present (background autofluorescence). (F) Trajectory of a representative single fluorescent dL5 molecule labelled with se-red-xc dye and imaged by stroboscopy (33.3 ms on to 99.9 ms off laser duty cycle) in chained cells of the *tat* mutant DADE. Shown are a bright field image of the cells (top left), fluorescence images at the indicated frame numbers, and the single molecule track coloured by frame number (bottom right). Scale bars = 2 µm. See also Video S1.

We assessed the ability of the two fluorogenic dyes to access different compartments of *E. coli* cells using strains in which dL5 was either expressed in the cytoplasm or targeted to the periplasm using the Tat signal sequence from the protein TorA (ssTorA). No background labelling of the cells was seen with either dye in the absence of dL5 (Figure 2A, top row).

The se-red-s dye was able to label both periplasmic dL5 (Figure 2A, 2nd row) and cytoplasmic dL5 (Figure 2A, 3rd row), indicating that this dye is fully cell permeable. The fluorescence observed with the periplasm-targeted dL5 was confined to the periphery of the cells consistent with dL5 being located in the periplasm in this strain (Figure 2A, B).

Periplasmic dL5 could also be labelled by the se-red-xc dye (Figure 2A). However, an analysis of the total photon count per cellular volume showed reduced labelling for se-red-xc in comparison to se-red-s (Figure 2E). Although the different substituents of the MG moiety do have some effect on the fluorescence properties of the activated dye,^27^ the rather large difference in fluorescence between the two dyes observed in this case indicates that se-red-xc is less permeable to the outer membrane (OM) than se-red-s.

When cells expressing dL5 in the cytoplasm were incubated with se-red-xc, the majority of cells exhibited no fluorescence (Figure 2A, 3rd row; Figure 2E) suggesting that the dye does not cross the inner membrane (IM). However, approximately 1 in 10 cells contained a single molecule of labelled dL5 (see for example the faintly fluorescent cell in the red box in Figure 2A, and Figure 2C,D). In contrast to the localisation pattern expected of a cytoplasmically-located protein, these rare fluorescent molecules were confined to the cell periphery (Figure 2B). The most plausible interpretation of this result is that these molecules correspond to dL5 proteins that have “leaked” from the cytoplasm to the periplasm. Consistent with this interpretation, the distribution of super-resolution localisations of these molecules resembles that of periplasm-targeted dL5 (detected with either dye) but is distinctively different from the central cytoplasmic localisations seen for cytoplasmic dL5 labelled with se-red-s (Figure 2B). To conclusively demonstrate that these peripherally-localized dL5 molecules are located in the periplasm, rather than being associated with the cytoplasmic face of the IM, we took advantage of the fact that *tat* mutants form cell chains in which the IMs of the individual cells are fully separated but their periplasmic compartments are contiguous.^28^ In this mutant background the cytoplasmically-expressed but se-red-xc-labelled dL5 molecules are mobile along the cell chains confirming that these molecules are located in the periplasm and not the cytoplasmic face of the IM (Figure 2F).

To summarize, our data show that the se-red-xc dye exclusively labels dL5 molecules located in the periplasm. Consequently, this dye/reporter combination is suitable for use as a topological reporter of protein exposure at the periplasmic side of the membrane. Surprisingly, our data also reveal a very low level of protein leak from the cytoplasm to the periplasm.

### Investigating the mechanism of dL5 leakage from the cytoplasm

**Figure 3:**
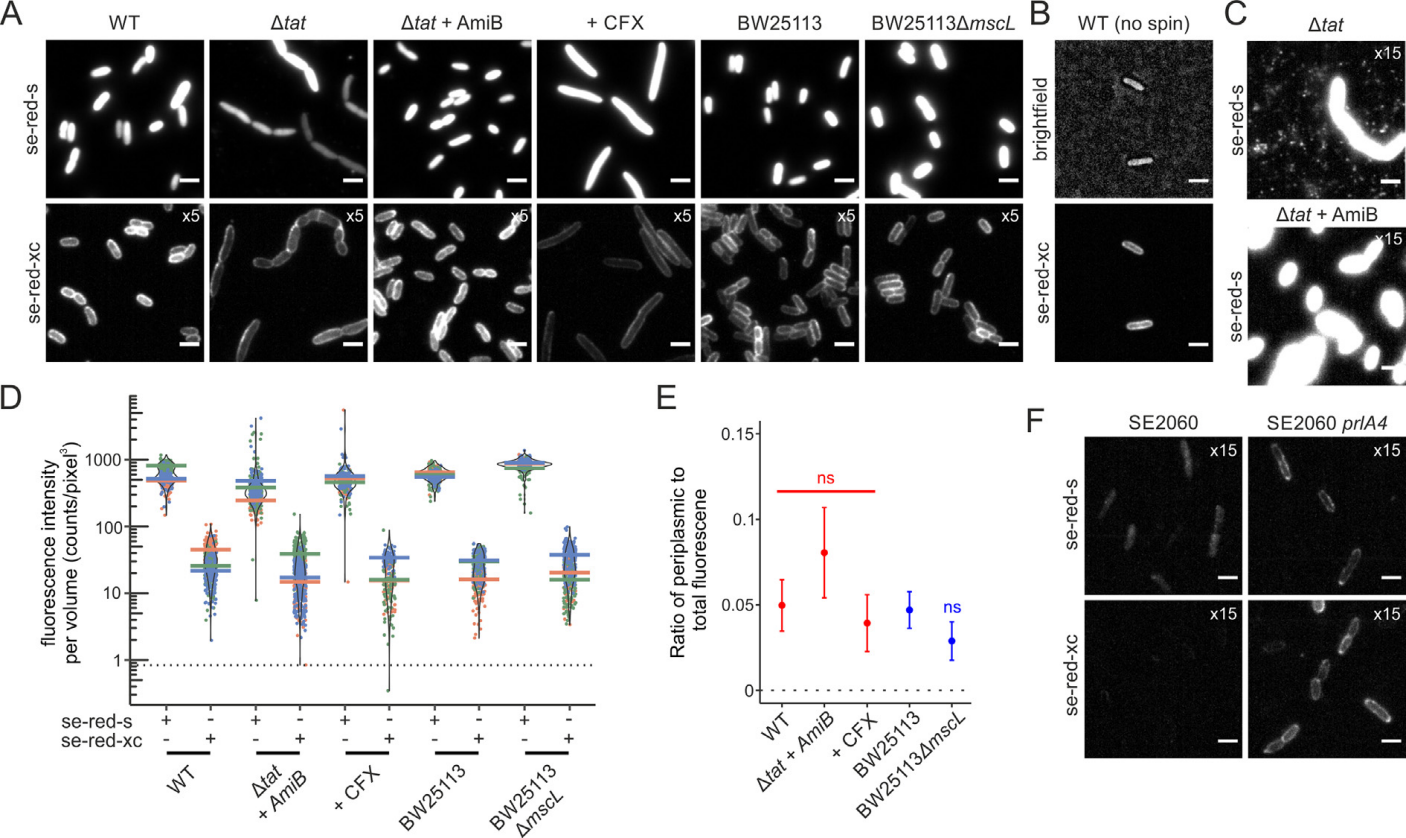
**Investigating the mechanism of dL5 leakage from the cytoplasm to the periplasm.** Strains expressed cytoplasmic dL5 from plasmid pQE-80 dL5 under inducing (A-E) or uninduced (F) conditions. The strains analysed were MC4100 (WT), the Δ*tatABC*Δ*tatE* derivative DADE (Δ*tat*), DADE overproducing AmiB to suppress cell chaining (Δ*tat* + AmiB), MC4100 treated with cephalexin for 20 min before induction of dL5 expression (+CFX), BW25113, BW25113 deleted for MscL (BW25113 Δ*mcsL*), SE2060, and SE2060 with mutations that allows Sec transport of secretory proteins with defective signal peptides (SE2060 *prlA4*). (A, B, C, F) Fluorescence images in the presence of the cell permeable dye se-red-s or cytoplasm-excluded dye se-red-xc. The images correspond to the maximum intensity Z-projection of 250 frames with brightness standardized except as indicated by multiplication signs. Scale bars = 3 μm. The image data are representative of cells from at least three independent cultures. (B) WT cells directly labelled with se-red-xc without centrifugation or change of medium. (C) dL5-containing outer membrane vesicles are shed by the *tat* null mutant DADE and vesicle production is suppressed by AmiB overproduction. (D) Distribution plots of the fluorescence intensity per cell volume from three biological repeats for each experimental condition for >100 individual cells per repeat. Each colour represents a biological repeat, each point is a single cell measurement, and the coloured horizontal bars represent the mean peak intensity of each biological repeat. The distribution of each full data set is shown by the violin plot. The dotted line is the mean intensity per volume of cells imaged with no dye present (background autofluorescence). (E) The background corrected ratios and standard errors of periplasmic fluorescence per volume (measured with se-red-xc) to total fluorescence per volume (measured with se-red-s) for the strains in D. An ANOVA with Welch correction (red) was used to compare the ratios of the MC4100-derived strains. A two-sided t-test with Welch correction (blue) was used to compare the ratios of the BW25113-derived strains. ns, non-significant. (F) Increased dL5 leakage in cells with a *prlA4* mutation.

dL5 is a heterologous protein without bacterial targeting signals. Thus, the observed leakage of this protein from the cytoplasm to the periplasm is likely to be a phenomenon that also affects other cytoplasmic proteins. This prompted us to investigate how such protein mislocalization occurs. To aid this analysis we increased the level of expression of dL5 such that every cell in the population now exhibits some molecules with mislocalization to the periplasm (Figure 3A, with the periplasmic localisation confirmed in chained cells in Figure S2).

To eliminate the possibility that manipulation of the cells for imaging caused dL5 to leak to the periplasm, we confirmed that dL5 mislocalization was still present in cells that were directly labelled in culture thus avoiding medium exchange, centrifugation, or resuspension (Figure 3B).

We initially examined three possible mechanistic explanations for the dL5 leakage: that it is due to a background level of non-specific protein export by the Tat system; that it occurs during the separation of the daughter cytoplasms during cell division; or that it occurs through transient opening of the large conductance mechanosensitive ion channel MscL, an event which appears to be responsible for protein leak from the cytoplasm under conditions of osmotic stress.^29^, ^30^, ^31^ After blocking each potential leakage pathway, peripherally localized fluorescence was still present in the cells (Figure 3A, bottom row, cephalexin (CFX) is used to block septal cell wall synthesis and thus prevent cell division) indicating that none of the proposed leakage mechanisms was solely responsible for the presence of dL5 in the periplasm. To determine whether these mechanisms nevertheless contribute to dL5 mislocalisation, we quantified periplasmic fluorescence. Cells without a Tat system have a perturbed cell envelope^28^, ^32^ which leads to shedding of OM vesicles (OMVs)^33^ and loss of periplasmic contents (Figure 3C) in addition to the previously reported phenotype of cell chaining that we exploited above.^28^ Because this OMV production would result in underestimating dL5 leakage to the periplasm, we corrected the envelope defect by overproducing the amidase AmiB as previously described^32^ (Figure 3C). No significant difference in periplasmic dL5 content was detected for any of the tested strains (Figure 3D). Thus, the leakage of dL5 to the periplasm is not due to the Tat system, cell division, or MscL.

We considered the possibility that dL5 leakage is due to adventitious signal sequence-independent transport by the Sec protein transport pathway. This proposal could not be tested using a null mutation because Sec is an essential function. Instead, we asked whether the dL5 leak is increased in cells carrying a *prlA4* allele. This strain allows the transport of secretory proteins with defective or absent signal peptides^34^, ^35^ through facilitating the opening of the Sec channel.^36^, ^37^, ^38^, ^39^ Strikingly, most of the dL5 protein in the *prlA4* strain can be seen to be located in the periplasm rather than the cytoplasm as dL5 has a peripheral localization even with the cell permeable se-red-s dye (Fig 3F). Thus, the mutant Sec apparatus is actively moving dL5 out of the cytoplasm. This shows that dL5 can be exported by the Sec system, at least when the specificity of the Sec translocon is relaxed. Although we cannot be certain that the native Sec system handles dL5 the same way, based on this observation we tentatively assign the dL5 leak to transport by the Sec apparatus.

### dL5 fusions to the C terminus of TatA, TatB, or TatC exhibit mixed topologies in live cells

The experiments described above demonstrate that binding of the dye se-red-xc by dL5 domains detects periplasmic localization with single-molecule sensitivity. We constructed strains that fuse the dL5 domain to the C terminus of the wild-type TatA, TatB, or TatC proteins. These strains were then labelled with se-red-xc to test our hypothesis that protein domains appended to TatA are transported from the cytoplasm to the periplasm.

The strains used in this work are named for the Tat proteins they produce. For example, the wild-type strain is termed ‘ABCE’ and possesses TatA, TatB, TatC, together with the TatA paralog TatE. TatA variants were expressed from the chromosome under the control of the TatA promoter. For technical convenience, TatB and TatC variants were expressed at approximately native levels from a low copy number plasmid in a Δ*tatBC* background, as in previous work, to give a strain designated 'AE pBC’.^12^, ^21^, ^24^ ‘d’, ‘h’, and ‘y’ designate fusion of the C terminus of the Tat component to, respectively, the dL5 domain, to a HaloTag (see later), or to YFP. A superscript R indicates that the fusion proteins contain a rigid α-helical linker (amino acid sequence (EAAAK)_8_AAA)^40^ between the Tat protein and the tag, as used in most previous imaging studies of TatA.^12^, ^20^, ^21^, ^24^ Thus ‘A^R^d’ is TatA fused to the N terminus of dL5 via a rigid linker domain.

We first confirmed that fusing a dL5 domain to TatA via a rigid linker sequence (TatA-[RL]-dL5) reproduces the inhibitory effect on Tat activity that was previously reported for analogous YFP fusions (strain A^R^dBC in Figure S1D).^22^ The dL5 fusion was also completely inactive even in the presence of the TatA paralogue TatE (strain A^R^dBCE in Figure S1D). Control experiments confirmed that the TatA-[RL]-dL5 fusion protein is stable and expressed at a similar level to wild-type TatA (compare strains A^R^dBCE and ABCE in Figure S1A).

**Figure 4:**
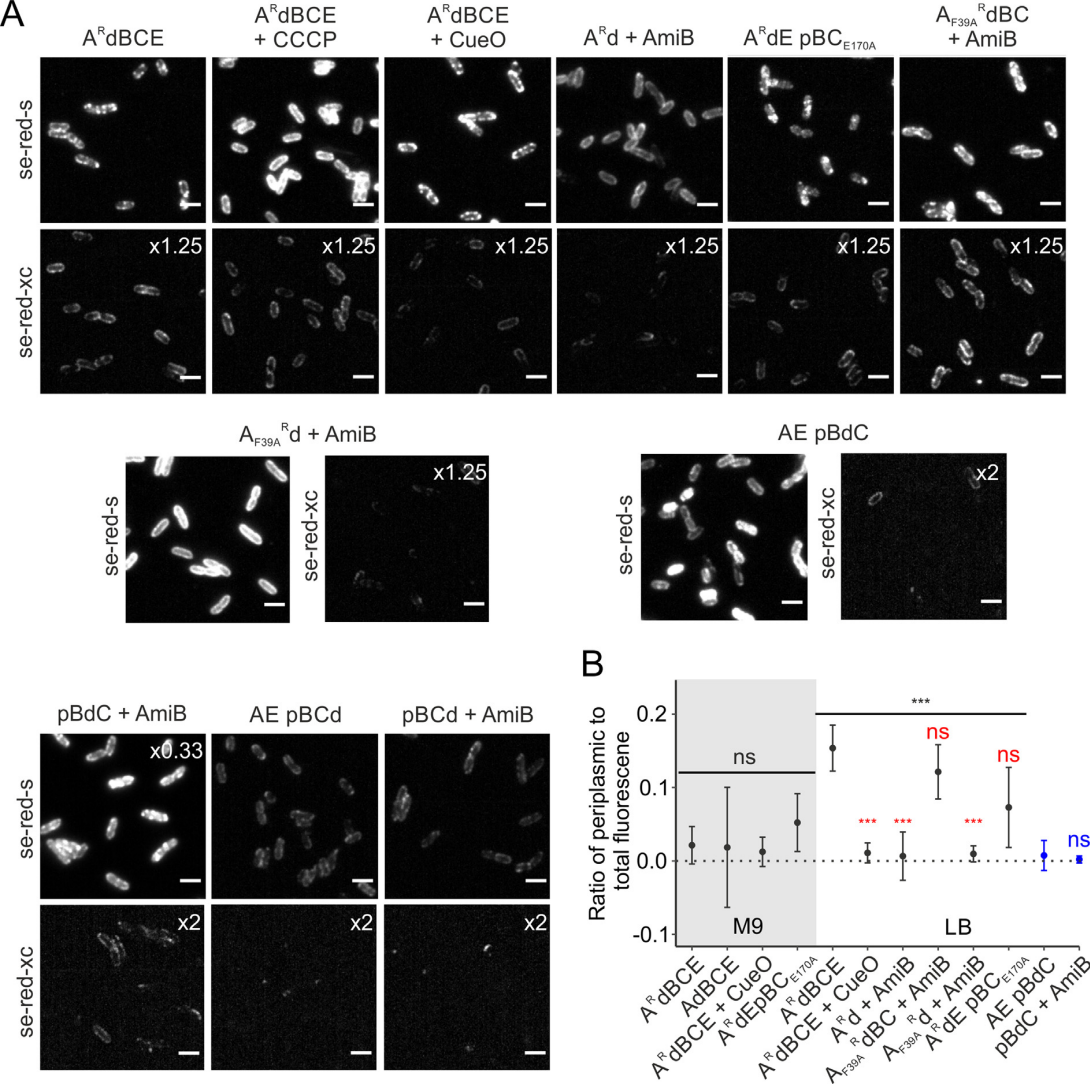
**Assessing the periplasmic exposure of dL5 fused to the C terminus of TatA, TatB, or TatC.** (A) Fluorescence images of the indicated strains grown in LB and imaged in the presence of either the cell-permeable se-red-s dye or the cytoplasm-excluded se-red-xc dye. The images correspond to the maximum intensity Z-projection of 250 frames with brightness standardized except as indicated by multiplication signs. Strains are named for the Tat proteins they produce with amino acid substitutions given as superscripts to the protein in which they are located, ‘p’ indicating that the proteins that follow are expressed at native levels from a plasmid, and ‘d’ and ‘^R^d’ indicating fusion to the N terminus of dL5 with or without a rigid linker sequence. For + AmiB strains, AmiB was overproduced from plasmid pSU18 AmiB (for strains A^R^d, A_F39A_^R^dBC and A_F39A_^R^d) or pSU40 AmiB (for strains pBdC and pBCd) to suppress cell chaining (phenotypes without AmiB are shown in Figure S3A). For + CueO cells, the Tat substrate CueO was overproduced from plasmid pQE-80 CueO by induction with 1 mM IPTG for 20 mins. For + CCCP images, the cells were treated with 50 μM of the protonophore CCCP for 1 min. Dyes were added to the cells following these procedures. The image data are representative of cells from at least three independent cultures. Scale bars = 3 μm. Analogous images for strains grown in M9 minimal medium are shown in Figure S3B. (B) Background corrected ratios and standard errors of periplasmic fluorescence per volume (measured with se-red-xc) to total fluorescence per volume (measured with se-red-s) for strains with dL5 fused to Tat components. The strains were cultured in either M9 medium (gray background) or LB medium (white background). Corresponding distribution plots for the raw fluorescence measurements are given in Figure S3C. An ANOVA with Welch correction was used to compare the fluorescence ratios of TatA-dL5 expressing strains grown on each medium (shown as a bracketing line with significance above). For TatA-dL5 expressing strains grown in LB medium pairwise comparisons to A^R^dBCE were then performed with a Games-Howell post-hoc test (results in red). A two-sided t-test with Welch correction (blue) was used to compare the fluorescence ratios for strains expressing TatB-dL5 fusions. ***p < 0.001. ns, not significant. In order to eliminate issues associated with the automated segmentation of chained cells the cell-chaining phenotype associated with inactive *tat* mutants was suppressed for cells grown in LB medium by AmiB overproduction. AmiB overproduction was unable to correct the chaining phenotype of cells cultured in M9 medium preventing the quantitative analysis of these strains.

Previous studies using TatA-YFP fusions have shown that the substrate-triggered assembly of TatA onto TatBC can be visualised in cells as the formation of bright fluorescent foci upon transport-saturating overproduction of a substrate protein^12^ (Figure 1C). Foci can also accumulate without substrate overproduction in strains where the Tat transport cycle is blocked after TatA assembly.^12^, ^20^, ^24^ The oligomerization behaviour of the TatA-[RL]-dL5 fusion was visualised using the cell-permeable se-red-s dye. Foci were apparent without substrate overproduction when TatE was absent (Figure S3A, B, strain A^R^dBC) but required substrate overproduction when TatE was present in M9 minimal medium, although not in rich LB medium (Figures 4A and S3A, strain A^R^dBCE). The foci were absent in cells treated with the protonophore carbonyl cyanide *m*-chlorophenyl hydrazone (CCCP) confirming their dependence on the cellular PMF. Taken together these observations show that the TatA-[RL]-dL5 fusion, like the analogous YFP fusion, is still capable of oligomerisation.

We proceeded to test the transport competition hypothesis by assessing whether the transport-inhibitory dL5 domain is exposed at the periplasmic side of the IM. Cells expressing the TatA-[RL]-dL5 fusion were incubated with the cytoplasm-excluded se-red-xc dye. Every cell exhibited peripheral fluorescence indicating that periplasm-localized dL5 domains were present (Figures 4A and S3A, strain A^R^dBCE). The TatA-[RL]-dL5 molecules giving rise to this fluorescence failed to oligomerize even after substrate overproduction (Figures 4A and S3A, strain A^R^dBCE + CueO) and are, thus, no longer able to respond to substrate activation of the Tat system.

Taken together our observations suggest that there are two sub-populations of the TatA-[RL]-dL5 fusion protein: one in which the C terminus is located in the cytoplasm (since it is stained with se-red-s but not se-red-xc) and still able to form oligomers, and one in which the C terminus of the protein is in the periplasm (and thus stains with se-red-xc) but which does not oligomerise. We cannot directly quantify the relative levels of the two subpopulations because the in-cell fluorescence of dL5 stained with our two dyes is not equivalent, as discussed above. Nevertheless, because most of the TatA-[RL]-dL5 molecules stained by se-red-s oligomerize, and are thus cytoplasm-exposed, we can infer that the periplasm-exposed subpopulation is only a very minor proportion of the total population. Periplasmic dL5 was still detected if the protonophore CCCP was added to the cells before dye labelling (Figure 4A, A^R^dBCE + CCCP stained with se-red-xc) indicating that the PMF is not required to maintain the observed periplasmic exposure of the dL5 domain. Periplasmic exposure of the dL5 domain was still seen if the rigid linker sequence was deleted from the TatA-[RL]-dL5 fusion (Figures S3A,D and 4B, compare strains A^R^dBCE and AdBCE) eliminating the possibility that the linker is responsible for the periplasm-facing orientation of the dL5 domain.

In the competition model for the low activity of TatA fusion proteins the observed periplasmic exposure of the dL5 domain of TatA-[RL]-dL5 would be interpreted as a successful attempt to translocate the fusion domain through the Tat system. This interpretation predicts that the periplasm-facing orientation of dL5 should depend on the activity of the Tat system. To test this prediction, we assessed whether periplasmic exposure of the dL5 domain was blocked by removal of the other Tat components (strain A^R^d), by the introduction of transport-inactivating substitutions (TatA F39A in the absence of the TatA paralogue TatE, or TatC E170A; strains A_F39A_^R^dBC and A^R^dE pBC_E170A_, transport activity characterised in Figure S1D), by a combination of these changes (strain A_F39A_^R^d), or by competition for transport from overproduction of an authentic Tat substrate (compare strains A^R^dBCE and A^R^dBCE + CueO in Figure 4A). None of these perturbations prevented TatA-[RL]-dL5 exposure in the periplasm, indicating that neither Tat transport nor the other components of the Tat system are absolutely required to achieve this topology (Figures 4A and S3A). To investigate whether these factors, nonetheless, influence the amount of periplasmic exposure of the dL5 domain, we quantified periplasmic fluorescence in the test strains as a proportion of total cellular TatA-[RL]-dL5 content (Figure S3D). This analysis showed significant decreases in the levels of periplasmic dL5 exposure in response to some, but not all, of the perturbations of the Tat system (Figure 4B). These significant differences were only seen for cells cultured in LB and not M9 medium, possibly because the level of periplasmic dL5 exposure in the parental strain is much higher when grown on LB. The decreases in periplasmic dL5 exposure were not consistently correlated to the transport activity of the strains (periplasmic exposure decreased in inactive strains lacking TatBCE but not in the presence of inactivating TatA F39A or TatC E170A substitutions), or ability to form TatA oligomers (periplasmic exposure decreases if assembly is blocked by removing Tat components, but also if assembly is induced by substrate overproduction), or other obvious feature of the strains.

Overall, our data suggest that periplasmic exposure of the dL5 domain of the TatA-[RL]-dL5 fusion can occur independently of the activity and components of the Tat system, but that the Tat system can also influence the extent of periplasmic exposure through a mechanism that is not linked to the activity of the Tat system in a straightforward way.

To assess whether transmembrane transfer of an appended dL5 domain is a specific feature of the TatA protein, or whether this phenomenon also occurs with other components of the Tat pathway, we characterized analogous C-terminal fusions of dL5 to the TatB and TatC proteins. Staining cells with the fully permeable dye se-red-s demonstrated that the TatB-dL5 and TatC-dL5 fusion proteins were successfully expressed and membrane-localized (Figure 4A, strains AE pBdC and AE pBCd) even though the TatC fusion could not be detected by immunoblotting (perhaps because recognition of the C-terminal peptide epitope of the antiserum is context-specific) (Figure S1C). As was observed for the TatA fusions, exposure of the dL5 domain to the periplasm was detected for both the TatB-dL5 and TatC-dL5 fusions (Figure 4A, B, strains AE pBdC and AE pBCd). This localization of the dL5 domain did not require Tat activity because the fusion proteins still bound se-red-xc in cells lacking TatA and TatE (Figure 4A, B, strains pBdC and pBCd).

Taken together, our results show that a fraction of the total cell population of both the single-pass membrane proteins TatA and TatB, and the multi-pass membrane protein TatC can locate their C terminus in the periplasm when that terminus is fused to the dL5 protein. Our results further show that this topological organisation does not require the presence of an active Tat transport system.

### An alternative fusion domain also detects mixed topologies for Tat proteins

The experiments above show that a subpopulation of TatA, TatB, and TatC proteins have a C-Out orientation when the C terminus is fused to a dL5 domain. To determine whether this behaviour depends specifically on the use of dL5 as the topological reporter we investigated the membrane orientation of Tat proteins that were instead fused to a HaloTag domain. HaloTag is a modified dehalogenase enzyme that forms covalent adducts with fluorophores bearing a long chain alkyl halide moiety (Halo Ligand).^41^

**Figure 5:**
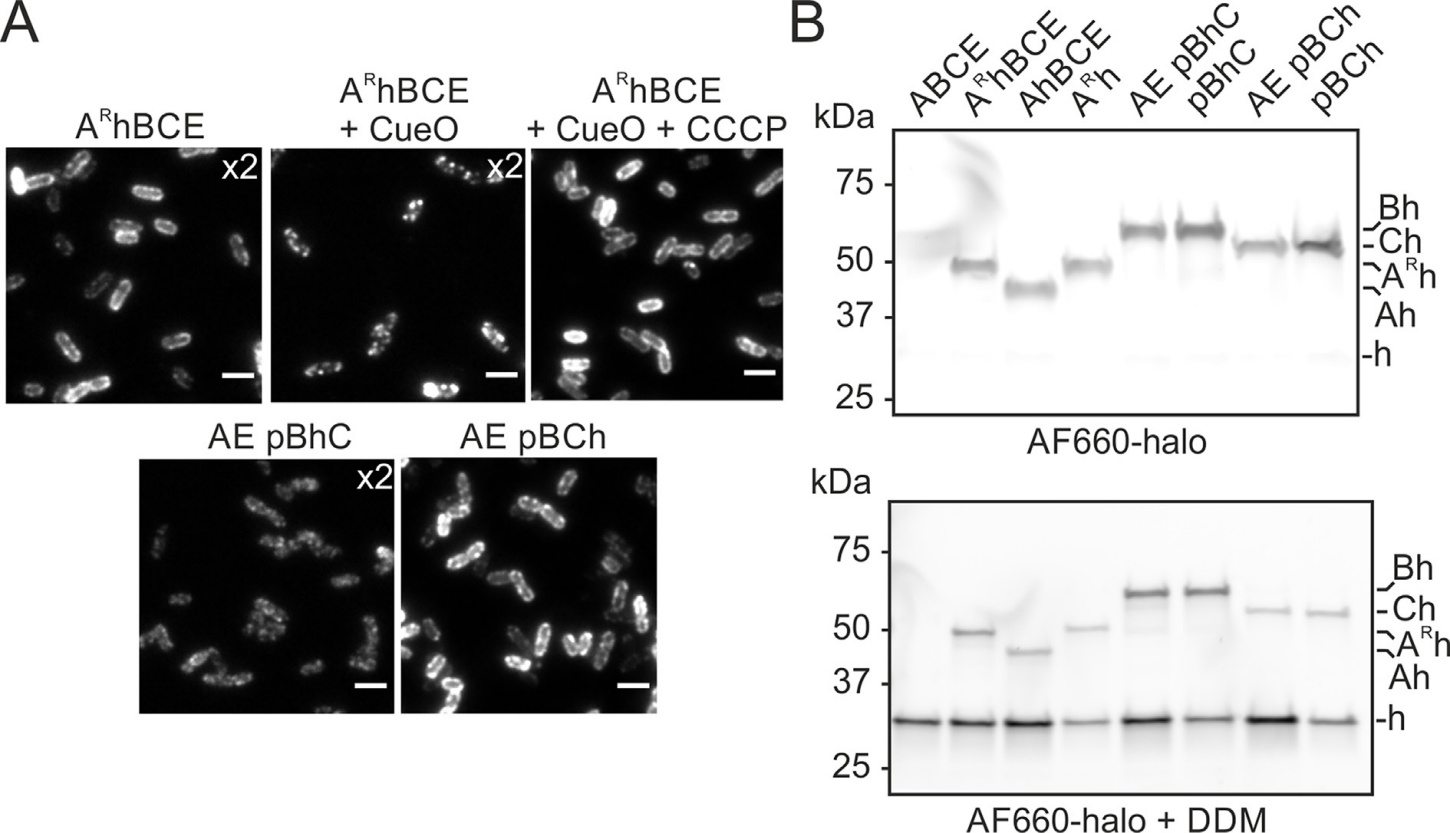
**Periplasmic exposure of HaloTag fused to the C terminus of TatA, TatB, or TatC.** (A) Single frame (33.3 ms exposure) fluorescence images of the indicated strains grown in LB medium and labelled with cell permeable JF646-halo. Strains are named for the Tat proteins they produce with ‘p’ indicating that the proteins that follow are expressed at native levels from a plasmid, and ‘h’ and ‘^R^h’ indicating fusion to the N terminus of HaloTag with or without a rigid linker sequence. In + CueO cells the Tat substrate CueO was overproduced from plasmid pQE-80 CueO by induction with 1 mM IPTG for 20 min before dye labelling. +CCCP indicates that after labelling and washing, the cells were imaged in the presence of 50 μM of the protonophore CCCP. The brightness is standardised across all images except where indicated by multiplication signs. The image data are representative of cells from at least three independent cultures. Scale bars = 3 μm. Analogous images for cells grown in M9 minimal medium are shown in Figure S4A and for strain AhBCE in Figure S4B. (B) The OM of the indicated strains was permeabilised and the cells either directly labelled with membrane-impermeable AF660-halo or labelled after membrane solubilisation with 1 % dodecyl maltoside (AF660-halo + DDM). The samples were then subject to SDS-PAGE and fluorophore-labelled proteins (identified to the right of the gels) were detected by in-gel fluorescence. All strains also expressed HaloTag (‘h’) in the cytoplasm to detect any dye entering that compartment. Note that due to the very different fluorescence levels the two gels are displayed at different brightness and contrast. Similar data were obtained for three repeats with independently grown cultures.

The Tat protein-HaloTag fusions we constructed were all stable and were expressed at levels that were equivalent to (TatA-HaloTag and TatB-HaloTag), or higher than (TatA-[RL]-HaloTag and TatC-HaloTag), that of the wild-type protein (Figure S1A–C). The phenotype of the TatA-[RL]-HaloTag fusion resembled that of the equivalent YFP fusion^12^ in that it was only able to support transport in the presence of TatE (compare strains A^R^hBCE with A^R^hBC in Figure S1D), and even this activity required the rigid α-helical linker between TatA and the HaloTag domain (compare strains A^R^hBCE and AhBCE in Figure S1D). The TatB-HaloTag fusion was completely inactive (strain AE pBhC in Figure S1D). However, the TatC-HaloTag fusion was able to support Tat transport (Figure S1D, strain AE pBhC). When cells expressing the HaloTag fusion proteins were stained with a cell permeable HaloTag ligand (Janelia Fluor 646 Halo ligand; JF646-halo) they exhibited peripheral fluorescence consistent with the fusion proteins being located in the cytoplasmic membrane (Figures 5A and S4). The oligomerization behaviour of the TatA-[RL]-HaloTag fusion again replicated the behaviour of the analogous YFP fusion^12^ in showing sensitivity to substrate overproduction and protonophore treatment only when TatE was also present (compare strain A^R^hBCE in Figure 5A with strain A^R^hBC Figure S4C).

In order to determine which side of the IM the HaloTag domain of the Tat fusion proteins is located, we developed methodology to selectively label periplasm-exposed HaloTags. In this method an osmotic shock is used to permeabilise the OM of the cell to allow entry to the periplasm of membrane-impermeant Alexa Fluor 660 HaloTag ligand (AF660-halo). HaloTag domains that are exposed at the periplasmic face of the IM are accessible to AF660-halo and are labelled. However, AF660-halo is unable to cross the IM and so HaloTag domains at the cytoplasmic side of the membrane are unlabelled. The cells are then subjected to SDS-PAGE and those Tat fusion proteins that have been labelled with AF660-halo are detected by in-gel fluorescence. To demonstrate that AF660-halo has not crossed the IM, each strain also expresses a plasmid-encoded HaloTag protein in the cytoplasm which would be labelled if the fluorophore has entered the cytoplasm.

Using this compartment-selective labelling strategy we detected periplasmic exposure of HaloTag fused to the C terminus of TatA (Figure 5B). This phenomenon was observed whether or not the fusion construct contained a rigid linker between TatA and the HaloTag (compare strains A^R^hBCE and AhBCE), whether or not the strain was active for Tat transport (compare transport active strain A^R^hBCE with transport inactive strains AhBCE and A^R^h), and whether or not other Tat components were present (compare strain A^R^hBCE with strain A^R^h). Labelling of the cytoplasmic HaloTag control was only detected if the cell membranes were first solubilized with detergent, confirming the specificity of the observed periplasmic labelling of the fusion proteins (Figure 5B). Exposure to the periplasm was also detected for HaloTag fused to the C-termini of TatB or TatC (Figure 5B, strains AE pBhC and AE pBCh) and this behaviour was unaffected by deleting both TatA and TatE from the strains (Figure 5B, strains pBhC and pBCh). HaloTag is, thus, detected at the periplasmic side of the membrane when fused to the C terminus of any of the three Tat components, and this orientation of the fusion domain does not require an active Tat system. This behaviour matches that of the equivalent dL5 fusions to the Tat proteins. Our labelling method does not allow us to determine what proportion of the total population of Tat proteins have their C terminus in the periplasm because the detergent treatment used to provide AF660-halo access to the cytoplasm markedly decreases labelling efficiency. However, the data in the next section suggest that the proportion of proteins with a periplasm-exposed C terminus is low, as was also observed for the dL5 fusions.

### The topological organisation of Tat proteins with periplasmic C-termini

The experiments described in the previous section establish that the C terminus of each of the Tat component-HaloTag fusion proteins can be exposed at the periplasmic face of the membrane (‘HaloTag-Out’) rather than exclusively adopting the accepted cytoplasmic localisation (‘HaloTag-In’). However, this analysis did not reveal how the remaining parts of each HaloTag-Out protein were oriented in the membrane. In order to more fully define the topology of the HaloTag-Out species, we combined periplasm-specific HaloTag labelling by AF660-halo with methoxypolyethylene glycol maleimide (PEG-Mal) accessibility labelling of cysteine residues introduced at positions throughout the protein sequence. PEG-Mal is a membrane-impermeable cysteine-reactive reagent that changes the electrophoretic mobility of the proteins it labels. It has previously been used to assess TatA and TatB exposure to the periplasm in OM-permeabilized *E. coli*.^16^ In our method, OM-permeabilized cells are labelled with both AF660-halo and PEG-Mal before being subjected to SDS-PAGE. In-gel fluorescence then reveals the PEG-Mal labelling patterns associated specifically with molecules with a HaloTag-Out orientation because only the molecules with this topology have been labelled with AF660-halo.

**Figure 6:**
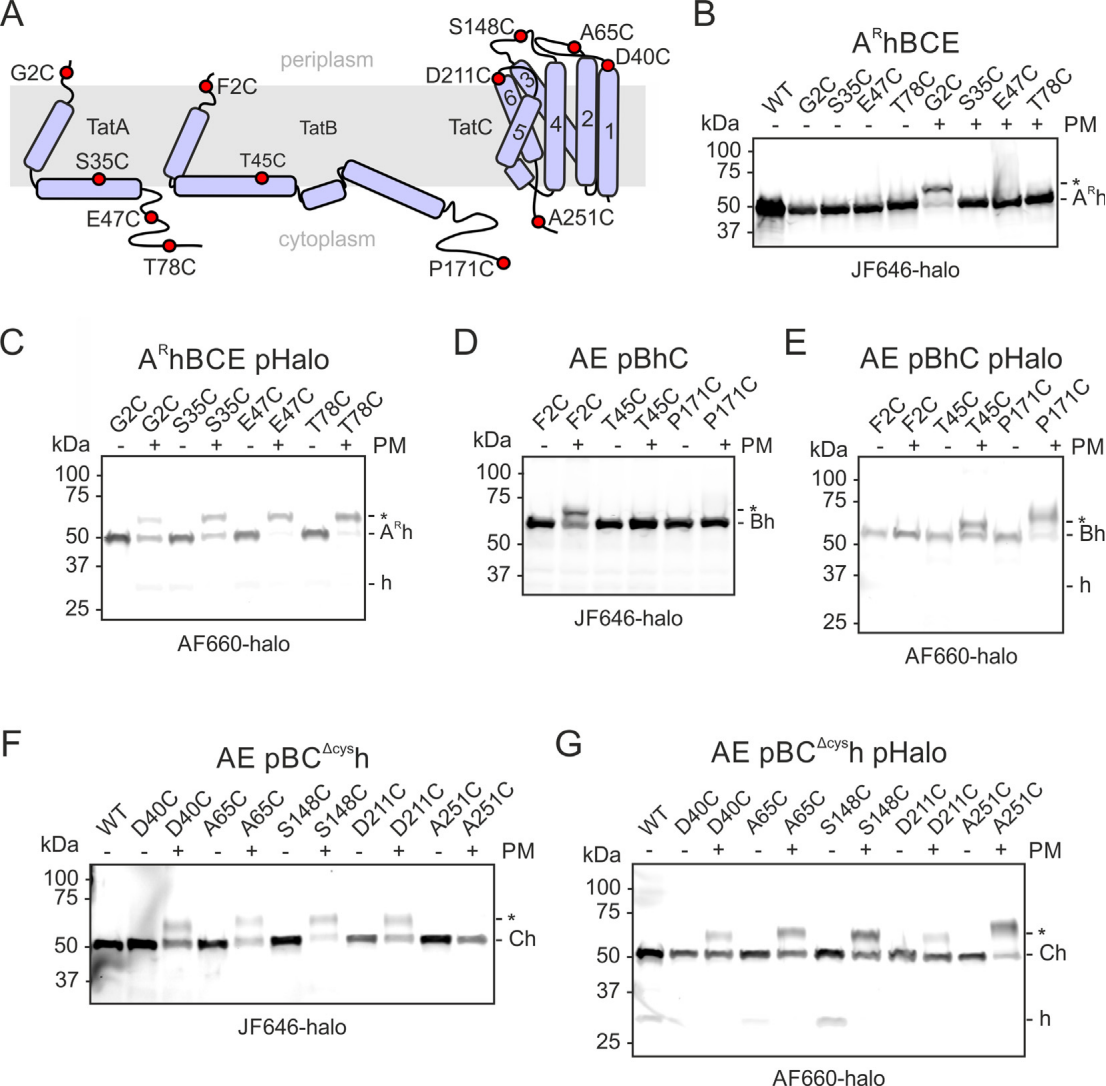
**The topological organization of TatA, TatB, and TatC molecules with a periplasmically located C-terminal HaloTag.** (A) Schematic showing the locations (red spheres) of the cysteine substitutions analysed here. The proteins are depicted in their normal topological organisation. (B–G) In-gel fluorescence of single cysteine variants of C-terminal HaloTag Tat fusions proteins labelled in OM-permeabilised cells with either (B, D and F) membrane-permeable JF646-halo to label all HaloTag fusion proteins in the cell, or (C, E and G) membrane-impermeable AF660-halo to selectively label molecules where the HaloTag is located at the periplasmic side of the membrane. The strains in (C, E and G) also express from a plasmid (pHalo) unfused HaloTag protein (expected migration position ‘h’) in the cytoplasm as a control for the compartment specificity of the AF660-halo labelling. Where indicated the OM-permeabilised cells were treated with PEG-Mal (PM) before dye labelling. Asterisks denotes PEG-Mal labelled adducts. Similar data were obtained for three repeats with independently grown cultures. Strains are named for the Tat proteins they produce with ‘p’ indicating that the proteins that follow are expressed at native levels from a plasmid, and ‘h’ and ‘^R^h’ indicating fusion to the N terminus of HaloTag with or without a rigid linker sequence. A TatC variant in which the native Cys residues were replaced by Ala residues was used (‘C_Δcys_’). Note that samples stained with the two different fluorophores exhibit very different levels of fluorescence and so are displayed at different brightness and contrast.

Single cysteine substitutions were introduced into the Tat-HaloTag fusions at topologically informative positions (Figure 6A). Strains expressing these proteins were labelled with the cell permeable HaloTag ligand JF646-halo to label all the copies of the protein that were present in the cell. Under these conditions the topological organisation of the proteins revealed by PEG-Mal labelling was in accord with the earlier PEG-Mal labelling study of TatA and TatB^16^ and with the crystal structure of TatC^19^ (Figure 1). Specifically, TatA variant G2C, TatB variant G2C, and TatC variants D40C, A65C, S148C, D211C showed mobility shifts consistent with PEG-Mal labelling and exposure to the periplasm, whilst the other single cysteine variants were unmodified (Figure 6A, B, D, F).

If, instead, AF660-halo was used to selectively label just those molecules with a HaloTag-Out domain, the pattern of PEG-Mal labelling changed. In the case of the TatB-HaloTag fusion those cysteine residues in the APH (residue 45) and C-tail (residue 171) now react with PEG-Mal indicating periplasmic exposure (Figure 6E). Conversely, the PEG-Mal labelling of the N terminus (residue 2) is lost suggesting that it now has a cytoplasmic location. Thus, the whole HaloTag-Out TatB molecule appears to have an inverted topology in the membrane, with the N terminus in the cytoplasm and all regions after the TMH in the periplasm. Broadly similar results were seen for the HaloTag-Out state of the homologous TatA protein where all cysteine residues that are normally in the cytoplasm are now labelled with PEG-Mal, showing that the APH and C-tail have changed location to lie at the periplasmic side of the membrane (Figure 6C; similar results were obtained with a fusion lacking the rigid linker, data not shown). However, labelling was still seen for the TatA N terminus (residue 2), albeit at a reduced level relative to that seen with the JF646-halo-detected proteins. This suggests that the HaloTag-Out TatA protein may have a mixed topology in which some copies of the molecule retain the original N-Out location of the N terminus but in other copies the TMH is fully inverted. Regardless of the behaviour of the N terminus of the molecule, the close to quantitative labelling of the C-tail residues by PEG-Mal shows that the full C-tail (and by inference the rigid linker sequence) of all HaloTag-Out TatA molecules is at the periplasmic side of the membrane.

In the HaloTag-Out form of TatC the C-tail of the protein (residue 251) shows a PEG-Mal-induced size shift as expected from the periplasmic location of the adjacent HaloTag domain, but in contrast to the normal cytoplasmic orientation of this part of the TatC molecule (Figure 6G). However, the cysteine residues located in the three periplasmic loops still react with PEG-Mal showing that TMHs 1–5 retain the topological organisation of the native TatC protein (Figure 6G). Taken together these observations indicate that both ends of TMH 6 are located in the periplasm and, thus, that TMH 6 is no longer membrane-spanning.

The relative labelling intensities observed with AF660-halo versus JF646-halo could not be used to quantify the proportion of the Tat-HaloTag fusions that have a HaloTag-Out orientation due to differences in the completeness of labelling obtained with the two dyes and because the fluorescent signal in the gel does not scale linearly with the amount of labelled protein. However, none of the PEG-Mal shifts associated with the HaloTag-Out orientation are detected in the experiments where the whole fusion protein population is labelled with cell permeable JF-646-halo. From this we infer that the HaloTag-Out orientation is only a very minor proportion of the total pool of each Tat protein, in agreement with the behaviour of the equivalent dL5 fusions.

## Discussion

### Tat transport is generically inhibited by C-terminal fusions to TatA or TatB

The starting point for this work was the observation that appending a FP to the C terminus of the TatA component of the Tat protein export system blocks Tat transport in the absence of TatE but does not prevent the oligomerization of TatA that is part of the normal transport process.^12^ Here we show that equivalent fusions of TatA to either a dL5 or HaloTag domain produce either the same effects as a FP fusion (in the case of a TatA-[RL]-Halo fusion) or have a still stronger effect on transport activity being transport-incompetent even in the presence of TatE (in the case of a TatA-[RL]-dL5 fusion) (Figures 4A, 5A, S1, S3A, S3B, S4). Thus, the phenotypic effects observed with the FP fusion appears general for TatA fusions to different folded domains, albeit that the domains tested do not vary greatly in hydrodynamic radius (34 Å, 23 Å and 23 Å for HaloTag, YFP, and dL5 respectively). We also find that fusing YFP or HaloTag domains to the C terminus of the TatA homologue TatB blocks Tat protein export (Figure S1; though note that the lack of activity of a TatB-YFP fusion observed in this work disagrees with a previous report^12^ and that we do not interpret the effects on transport of the equivalent dL5 fusion because TatC expression is affected in this strain). By contrast, the C terminus of TatC was tolerant to the addition of fusion domains.

Taken together, our data indicate that appending a C-terminal domain is generically inhibitory for TatA homologues. This suggests that the domains appended to TatA and TatB likely exert their inhibitory effects on transport by the same mechanism. Any model to explain the inhibition phenomenon must take this new criterion into account. Mechanistic similarity would be plausible under the transport competition hypothesis in which the appended domains are envisaged to engage with the translocation apparatus in place of the substrate proteins. This is because for both TatA and TatB the fusion domains are anchored to the translocation machinery by long flexible regions of polypeptide that would allow access to the transport pathway (Figure 1A). However, the experimental testing that is discussed next provides no support for the competition hypothesis and so alternative models of inhibition such as steric hinderance need to be considered.

### Detection of abnormal membrane protein topogenesis

Although all three Tat proteins would normally have a C-in orientation across the cytoplasmic membrane, we found that both dL5 and HaloTag domains could be detected at the periplasmic side of the cytoplasmic membrane when fused to the C terminus of any of TatA, TatB, or TatC. In all cases the molecules displaying this C-Out orientation were a very small proportion of the total population of fusion proteins present in the cells. The presence of such periplasmically-exposed fusion domains would be consistent with the transport competition hypothesis. However, whilst the hypothesis predicts that the fusion domains should only be found in the periplasm as a consequence of Tat transport, we found that periplasmic exposure occurred even when the Tat system was inactivated by transport-blocking amino acid substitutions or by removing pathway components. This is particularly noteworthy in the case of fusions to TatB and TatC where the C-Out topology was still observed in the absence of the TatA protein even though the TatA protein is responsible for the physical movement of Tat substrates across the membrane. Additionally, site-specific labelling experiment showed that the C-Out TatA and TatB HaloTag fusion proteins had experienced complete, or close to complete, topology inversion. This observation is surprising if the fusion domain has undergone Tat transport since it might be expected that only the HaloTag and immediately adjacent part of the TatA polypeptide and linker would need to move across the membrane. Taken together, our assessments of periplasmic exposure imply that the fused protein domains do not reach the periplasm as a consequence of Tat activity and thus our data fail to substantiate a key prediction of the competition hypothesis. Instead, the observed population of C-Out proteins is likely a consequence of errors in protein topogenesis during biosynthetic insertion of the proteins into the membrane. To the best of our knowledge, this is the first report of the detection of such errors for native proteins which have not had their topological signals altered. Whilst examples are known of proteins that have two physiologically relevant topologies established at the translocon (e.g.,^42^, ^43^ and some weakly hydrophobic TMHs in multispanning proteins can alter topology during the biogenesis process (reviewed in 5) neither of these scenarios are relevant for TatA and TatB which are single spanning proteins with only one functionally relevant topology (Figure 4A).

Membrane protein insertion in *E. coli* normally occurs co-translationally. The signal recognition particle (SRP) recognises the first TMH of the protein as it emerges from the ribosome and then targets the ribosome-nascent chain complex to the SecYEG translocon and/or the YidC insertase for integration into the cytoplasmic membrane.^3^ Biogenesis of the Tat proteins is likely to be typical in this respect because all three proteins have been shown to recruit SRP to ribosomes *in vivo*^44^ and, in the case of TatC, membrane integration has been demonstrated to involve SRP and both SecYEG and YidC.^45^, ^46^ The topology of membrane proteins is determined in accordance with the positive-inside rule. This topological signal is very strong for TatA and TatB as there are no positively charged residues at the N-terminal side of the TMH (and the initial fMet residue of these proteins is apparently unmodified and thus uncharged^47^) but either five (TatA) or four (TatB) basic residues in the APH (Figure 1). Thus, insertion in the canonical C-In orientation is expected and our observation of a population of fusion proteins with the APH and following regions in the periplasm violates the positive-inside rule and is a failure of correct topogenesis.

One possible explanation for the incorrect topogenesis of the TatA and TatB fusion proteins is that during the membrane insertion process the TMH explores both orientations across the membrane, but folding of the appended reporter domain traps the protein in its current orientation before the correct topology can be resolved. However, we regard this possibility as unlikely because it implies that the topology of the TMH can still be undecided by the stage at which the whole soluble part of the fusion protein (corresponding to 468 amino acids in the case of the TatB-HaloTag fusion) has been threaded through the translocation channel. In the corresponding situation with the wild type protein, it would require the translocon to be capable of returning the entire soluble domain (corresponding to 161 amino acids in the case of TatB) from the periplasm to the cytoplasm to resolve TMHs that initially adopt a C-Out orientation. Both these scenarios are unlikely. Note, also, that the fusion domain is sufficiently well-separated from the TMH that it is not expected to directly affect the topogenesis of the TMH since this relies on the flanking charges. In summary, our observations indicate aberrant topogenesis for a sub-population of TatA and TatB molecules.

TatA and TatB are monotopic membrane proteins with extrinsic domains only on the cytoplasmic side of the membrane. We also found that a C-terminal fusion domain is mislocalised in a small subpopulation of the multi-spanning protein TatC. However, it is only the final TMH that has the incorrect topological organisation and so in this case it is quite plausible that folding of the fusion domain has trapped a transient state in topogenesis during which the TMH visits a non-native orientation.

Cells have quality control systems to remove aberrantly folded membrane proteins.^48^, ^49^, ^50^ However, the topological defects in the Tat proteins identified here are expected to escape these correction mechanisms because they do not cause incorrect folding. More generally, many experimental studies have stably expressed topologically-defective variants of multispanning membrane proteins in *E. coli* (e.g. 51) suggesting that post-insertional membrane protein quality control is weak in bacteria and thus that the rate of incorrect protein topogenesis has to be kept low, as we observe.

### Cytoplasmic proteins can leak into the periplasm

In the course of this work we made the unexpected observation that cytoplasmically expressed dL5 protein leaks at a very low level to the periplasm. To the best of our knowledge this is the first definitive evidence of protein leak across the cytoplasmic membrane in unperturbed cells. Two particular characteristics of our imaging methodology have allowed this discovery. First, periplasmic localisation is established in intact cells (using two independent methods, namely dye accessibility and single-molecule distributions in single and chained cells). This avoids the possibility of low-level cross-contamination between compartments that arises with classical subcellular fractionation methods. Second, our method has single-molecule sensitivity which allows the detection of even minimal rates of leakage. The mechanism that results in the leak of dL5 should be non-selective as dL5 is a heterologous protein that contains no specific bacterial targeting information. Consequently, it is likely that other cytoplasmic proteins also leak to the periplasm.

Multiple previous studies have reported that cytoplasmic proteins can be detected outside Gram-negative bacteria.^52^, ^53^ This protein release has often been ascribed to either cell lysis or to the transient release of cell contents on osmotic shock.^52^, ^54^ In the latter case the large conductance mechanosensitive ion channel MscL has been implicated in mediating initial protein leak across the IM.^29^, ^30^, ^31^ However, osmotic challenge does not appear to be responsible for the re-localization of dL5 to the periplasm that we observe here since this still occurs in the absence of the MscL protein or when the medium surrounding the cells is kept constant in our experiments. Our data also rule out non-specific movement through the Tat pathway or leak during cell division as explanations for this phenomenon. We were, however, able to detect high levels of dL5 export in a mutant that reduces the dependence of Sec channel opening on secretory protein targeting signals.^36^, ^37^, ^38^, ^39^ Thus, the Sec apparatus is inherently capable of exporting dL5. This observation does not prove that the native Sec apparatus is responsible for the dL5 leak but would be consistent with this hypothesis. Further studies will be required to substantiate this proposal.

## Materials and Methods

### Genetic constructs

The strains used in this work are listed in Table S1, the plasmids in Table S2, and the primers in Table S3. All *tatA* fusions were integrated at the *att* site and all *tatB* and *tatC* alleles were expressed from plasmid p101C*TatBC.^12^ See *SI Materials and Methods* for details.

### Fluorescence microscopy

Cells containing dL5 constructs were stained with 100 nM se-red-s or se-red-xc (Sharp Edge Labs). Cells expressing HaloTag fusion proteins were treated with 1 μM Janelia Fluor 646 HaloTag ligand (Promega) for 20 min at 37 °C and washed four times before imaging. Cells were imaged on agarose pads by highly inclined and laminated optical sheet (HiLo) illumination using a Nanoimager (Oxford Nanoimaging) equipped with a 640 nm 1 W DPSS laser. See *SI Materials and Methods* for experimental and data processing details.

### In-gel fluorescence of labelled HaloTag fusion proteins

The OM of the cells was permeabilised as described.^55^ Where required cells were labelled for 40 min with 5 mM PEG-Mal (Sigma). The cells were then incubated for 20 min at 25 °C with 100 nM Alexa Fluor 660 HaloTag Ligand (Promega) to label HaloTag proteins located in the periplasm followed by a 10 min incubation with 0.05 % (v/v) 1,3-dibromopropane (Sigma) to block the labelling of all remaining HaloTag proteins in the cell. The samples were subjected to SDS-PAGE and in-gel fluorescence visualised using an Amersham Typhoon 5 Biomolecular Imager equipped with a 635 nm laser and 670 ± 30 bandpass filter. See *SI Materials and Methods* for details.

## Author contributions

SJH conducted all experimental work except for the collection of dL5 migration data in chained cells which was carried out by AB. HLM wrote the programmes for image intensity analysis and assisted with the statistical analysis. The work was conceived by SJH, ANK, and BCB. All authors contributed to the writing of the manuscript.

## CRediT authorship contribution statement

**Samuel J. Hickman:** Conceptualization, Methodology, Validation, Formal analysis, Investigation, Writing – original draft, Writing – review & editing, Visualization. **Helen L. Miller:** Software, Formal analysis, Writing – original draft. **Alfredas Bukys:** Validation. **Achillefs N. Kapanidis:** Conceptualization, Writing – review & editing, Supervision, Funding acquisition. **Ben C. Berks:** Conceptualization, Writing – original draft, Writing – review & editing, Supervision, Funding acquisition.

## Declaration of competing interest

The authors declare the following financial interests/personal relationships which may be considered as potential competing interests: ‘Author Achillefs Kapanidis has a financial interest in Oxford NanoImager which manufactures the fluorescence microscope used in this work. Author Achillefs Kapanidis is a member of the Editorial Board of the Journal of Molecular Biology.’

## Data Availability

Custom written Matlab code is available online at https://github.com/HLMiller-Imaging/NIM-Total-Intensity-Analysis.
