# Supplementary material for: Aberrant Topologies of Bacterial Membrane Proteins Revealed by High Sensitivity Fluorescence Labelling
Source: J Mol Biol. 2024 Jan 15;436(2):168368. doi: 10.1016/j.jmb.2023.168368 (PMC11867995; doi:10.1016/j.jmb.2023.168368)
Supplement: Supplementary data 1 [file mmc1.pdf]

Supplemental Information

Aberrant topologies of bacterial membrane proteins revealed by high sensitivity fluorescence labelling

Samuel J. Hickman, Helen L. Miller, Alfredas Bukys, Achillefs N. Kapanidis, and Ben C. Berks.

Supplemental Material and Methods

Growth conditions

Unless otherwise indicated, cells were cultured in LB medium [1] at 37°C and 180 rpm shaking. Antibiotics were used at the following concentrations: ampicillin (100 µg /ml), kanamycin (50 µg/ml), chloramphenicol (25 µg/ml), cephalexin (60 µg/ml).

Genetic constructs

All *tatA* fusions were created starting from pBSTatAry [2], which contains a P_{tatA}-*tatA-RL-eyfp*^{A206K} cassette in pBlueScript KS (Stratagene). *eyfp* was excised from pBSTatAry using BsiWI and BamHI and replaced either with the *dL5* gene amplified from pET21-10XHis-GST-HRV-dL5 [3] using primers dL5F and dL5R or with the *halotag* gene amplified from pHTC HaloTag® CMV-neo Vector (Promega) using primers HaloF and HaloR, creating plasmids pBSTatArdL5 and pBSTatArhalo respectively. To remove the rigid linker (RL) coding sequence Q5 site directed mutagenesis (NEB) was used with primers NORLdL5F and NORLdL5R for pBSTatArdL5, and NORLhaloF and NORLhaloR for pBSTatArhalo, creating pBSTatAdL5 and pBSTatAhalo respectively. Point mutations were introduced by Q5 site directed mutagenesis. All *tatA* alleles were inserted into the *E. coli* phage lambda attachment site (*att*) by subcloning as an EcoRI-BamHI fragment into plasmid pRS552 [4] and then using the resulting plasmid to transform the desired background strain as described [5].

A plasmid to express a TorA signal sequence-dL5 fusion was constructed as follows. *sstorA-gfpmut2* from plasmid pTGS [6] was amplified using primers TGF and TGR and cloned between the BseRI and BamHI sites of pQE-80 (Qiagen) to create pQETG. The XbaI site between *ssTorA*

and *gfpmut2* was changed to a BsiWI site using Q5 site directed mutagenesis with primers TGBsiWIF and TGBsiWIR to create pQET$_{BsiWI}$G. Finally, the *dL5* gene lacking a start codon was amplified from pET21-10XHis-GST-HRV-dL5 using primers BsiWIdL5F and dL5BamHIR and used to replace the *gfpmut2* containing BsiWI-BamHI fragment of pQET$_{BsiWI}$G to produce plasmid pQE-80 ssTorA-dL5.

To produce plasmid pQE-80 dL5 that expresses the dL5 domain without a signal peptide, Q5 site directed mutagenesis using primers delTF and delTR was used to remove the signal peptide coding region from pQE-80 ssTorA-dL5.

A plasmid to express HaloTag was constructed as follows. An AgeI restriction site was inserted between *sstorA* and *gfpmut2* in pQETG using Q5 site directed mutagenesis with primers TGAgeIF and TGAgeIR producing plasmid pQET$_{AgeI}$G. The *halotag* gene was amplified from pHTC HaloTag® CMV-neo Vector using primers AgeIhaloF and haloBamHIR and used to replace the *gfpmut2*-containing AgeI-BamHI fragment of pQET$_{AgeI}$G. The signal sequence coding region and AgeI restriction site were then removed using Q5 site directed mutagenesis with primers delTF and delThaloR creating pQE-80 HaloTag.

Plasmids to express TatBC at close to native levels with a HaloTag or dL5 domain fused to the C-terminus of either TatB or TatC were constructed using two fragment Gibson assembly (NEB). p101C*TatBC [2] was used as the template for the vector fragment, and pHTC HaloTag® CMV-neo Vector or pET21-10XHis-GST-HRV-dL5 as targets for the amplification of the *halotag* or *dL5* genes, respectively. Where a rigid linker coding sequence was included in the construct, the vector amplification target was pBSTatArdL5 for the *RL-dL5* fragment or pBSTatArhalo for the *RL-halotag* fragment. Point mutations were introduced into the plasmids by Q5 site directed mutagenesis. Gibson assembly was used to generate plasmids expressing HaloTag fusions to cysteine-less or single cysteine variants of TatC. p101C*TatBCh

was used for the vector fragment template and plasmids pTAT101 Cys-less [7], pTatBC Cys-less A251C, or pTAT101-cys-less derived plasmids encoding TatC with the single amino acid substitutions D40C, A65C, S148C, or D211C were used as the templates for the *tatC* mutant alleles.

To render AmiB expression from plasmid pSU18-AmiB [8] independent of LacI repression the *lacO* site was removed using Q5 site directed mutagenesis with primers D_lacO_F and D_lacO_R producing plasmid pSU18-AmiB-*lacO*.

All constructs were verified by sequencing.

Fluorescence microscopy of dL5 and HaloTag fusion proteins

Strains expressing dL5 and HaloTag fusion proteins were grown from a single colony for 16 h at 37 °C and 180 rpm shaking in either M9 minimal medium comprising M9 salts [9], 0.1 % (w/v) tryptone, 0.2 % (w/v) glucose, 2 mM $MgSO_4$ and 0.1 mM $CaCl_2$, or LB medium. To obtain populations of single cells for those strains that would normally exhibit a cell chaining phenotype due to loss of Tat function the strain was transformed with the AmiB-overproducing plasmids pSU18 AmiB-*lacO* (for DADE pQE-80 dL5), pSU18 AmiB (for strains A^Rd, $A_{F39A}{}^R$dBC and $A_{F39A}{}^R$d), or pSU40 AmiB (for strains pBdC and pBCd) and cultured in LB medium (we empirically determined that the plasmid was unable to suppress the chaining phenotype in minimal medium). Where preservation of the chaining phenotype was desired, strains were grown and imaged in LB. 100 µl of the 16 h cell culture was sub-cultured into 5 ml of the same prewarmed medium and grown to mid-exponential growth phase (OD_{600} = 0.5) at 37 °C and 180 rpm shaking. For strains designated in the text as producing high levels of dL5 from an expression plasmid or producing high levels of Tat substrate protein CueO from an expression plasmid, 1 mM IPTG was added to the culture for 20 min (for dL5

induction) or 40 min (for CueO induction) before cell harvesting. Strains designated as producing low levels of dL5 were left uninduced. 1 ml of the cell culture was then centrifuged at 4,000*g* for 2 min to harvest the cells.

Cells containing dL5 constructs were washed twice by resuspension and re-centrifugation with 1 ml of imaging buffer (M9 salts, 0.2 % (w/v) glucose) and then resuspended in 100 µl imaging buffer. se-red-s (alternative name MG-Ester [10]) or se-red-xc (alternative name MG-B-Tau [3])(both Sharp Edge Labs) were added to a final concentration of 100 nM and incubated with the cells for 5 min at 25 °C.

For strains expressing HaloTag fusion proteins, Janelia Fluor 646 HaloTag ligand (Promega) was added to a final concentration of 1 µM and incubated with the cells for 20 min at 37 °C with shaking. The unreacted ligand was removed by four wash steps (resuspension and re-centrifugation with 1 ml of imaging buffer) and the cells were then resuspended in 20 µl imaging buffer.

Labelled strains were directly spotted onto 1 % low-fluorescence agarose (Biorad) pads containing imaging buffer. The glass coverslips (0.17 mm thickness) (Menzel-Glaser) used for imaging were pretreated by heating to 500 °C in a furnace for 1 h to remove fluorescent background particles. Brightfield and fluorescence images were acquired at 25 °C using a Nanoimager (Oxford Nanoimaging) equipped with a 640 nm 1W DPSS laser. Optical magnification was provided by a 100 × oil-immersion objective (Olympus, numerical aperture (NA) 1.4) and images were acquired using an ORCA-Flash4.0 V3 CMOS camera (Hamamatsu) with a pixel size of 117 nm. The focal plane was manually positioned to the central cross-section of the cells using brightfield illumination. Fluorescence images were routinely collected using highly inclined and laminated optical sheet (HiLo) illumination, with a 33.3 ms (30 Hz) exposure time, and 15 % laser power (640 nm).

Super resolution fluorescence localisations were performed using the Nanoimager software (version 1.7.3) using the default settings.

Quantification of fluorescence intensity

To quantify the mean fluorescence intensity per unit volume the bacteria were first segmented using Fiji [11]. Brightfield images were converted to a binary mask by a 10-pixel rolling background subtraction, followed by a default auto-threshold. Small gaps in the cells binary mask were closed using the 'fill holes' operator, and small regions of background signal were removed using the 'open' operator. Cells were then segmented by the watershed algorithm [12].

Next, the Fiji plugin MicrobeJ [13] was used to extract the mean intensity per cell, intensity versus time trace for each cell, and cell dimensions. The following MicrobeJ segmentation filters for rod-shaped cells were used: area 100-500 pixels^2, pole-to-pole length (L) 10-40 pixels, width (W) 9.5-15 pixels, curvature 0-0.1 and angularity 0-0.25. Cell volumes (V) were calculated from the extracted cell dimensions assuming a model of a cylindrical cell with hemispherical endcaps according to equation 1:

$$V = \frac{\pi W^2}{12}(3L - W) \qquad (1)$$

The fluorescence intensity measurements were corrected for laser turn on effects associated with the Nanoimager using custom MATLAB scripts as detailed in the Supplementary Methods. The corrected fluorescence intensity was then divided by the volume of the cell found from the MicrobeJ outputs. Kernel density estimates of the

distribution of the fluorescent counts per unit volume across the cell population were made, and a Gaussian distribution with fixed centre was fitted to the primary peak of the distribution. This Gaussian fit returns the mean and standard deviation of fluorescent counts per unit volume across the cell population.

For statistical comparison of the ratios of periplasmic signal between strains, the primary peak periplasmic intensity per volume distribution (se-red-xc signal) was divided by the primary peak total intensity per volume distribution (se-red-s signal) for each biological repeat with a background subtraction of the peak intensity of strains containing no dL5 imaged with se-red-s or se-red-xc as appropriate. Each biological repeat corresponds to all the se-red-s and se-red-xc intensity data collected on the same batch of cells. The Welch One-Way ANOVA Tests and Games Howell Post-hoc Tests were performed using the R package rstatix (version 0.7.0) (https://rpkgs.datanovia.com/rstatix/). Welch modified two-sample t-tests were performed using the R package BSDA (version 1.2.0) (https://alanarnholt.github.io/BSDA/).

Correction of fluorescence data for laser turn on effects

The Nanoimager laser is turned on to start each image acquisition. The laser intensity stabilises over a few seconds, with the stabilisation time varying for each acquisition. The associated increase in background autofluorescence has a substantial effect on the measured cellular fluorescence of low fluorescence samples and must be corrected for (Fig. S5).

Using custom written Matlab code (available online at https://github.com/HLMiller-Imaging/NIM-Total-Intensity-Analysis), the laser turn on parameters were determined by fitting the average pixel intensity $I_B(t)$ in a 40 x 40 pixel background region over time using equation 2:

$$I_B(t) = a - be^{-\alpha t} \qquad [2]$$

Where *a* is the constant background intensity count achieved with stable laser emission, *b* is the maximum deviation from the constant background intensity count, and α is the fitted exponential constant governing the laser intensity stabilisation in this image.

The individual cell intensity versus time traces in each image were fitted taking into account both photobleaching and laser turn on (which increases fluorescence background intensity up to the constant level and increases fluorescence emission proportional to the increasing laser illumination power) by using equation 3 for the total fluorescence intensity of the cell over time, *I(t)*:

$$I(t) = (1 - Ae^{-\alpha t})\left(B + Ce^{-\beta t}\right)$$

[3]

C is the parameter of interest and is the fluorescence intensity due to the labelled molecules. This fluorescence decays over time with photobleaching decay constant *β*. B is the fluorescence background had the experiment been performed under constant laser illumination. *α* is the exponential constant governing the laser intensity stabilisation obtained from fitting equation 2 to the fluorescence background. *A* is the proportion of laser power which is not available when the laser is first turned on.

In fitting equation 3 we constrained parameter values within reasonable limits to avoid physically unrealistic solutions. No fitted parameter could be less than zero. Some values in the fit to equation 3 were constrained with the values found from fitting equation 2 to the

background. Comparing equations 2 and 3 gives $A = {}^{a}/_{b}$, and $B = a$. Allowing a small range to account for the errors in the background fitting and the autofluorescence of the cells, the values of A and B were constrained to be in the ranges: ${}^{a}/_{5b} < A < {}^{5a}/_{b}$ and ${}^{a}/_{2} < B$; the upper limit on *B* is set to *2a* unless the fluorescence signal exceeds 10^5 counts, at which point it is raised to *5a* to account for stray fluorescence from other nearby, high intensity cells. The photobleaching decay constant of the fluorophore, *β*, was measured independently and found be of order seconds, with small variation dependent on the environment. In the low fluorescence signal samples the stepwise photobleaching of individual fluorophores can be seen; to stop the step edges being fitted with fast photobleaching decays, a limit corresponding to a minimum mean lifetime of 0.66s was set for all traces. When the fluorescence signal from the labelled molecules is large compared to the background fluorescence, the correction due to laser turn on is small and the measured fluorescence intensity of the cell, C, was constrained to three times the maximum height above the background in the intensity versus time trace to avoid unphysical solutions. For the same reason, C was constrained to the maximum intensity difference of the raw trace in samples where the background fluorescence increase was larger than the fluorescence signal. In these fits we do not consider the higher order correction due to the proportionality of the photobleaching rate and laser illumination intensity.

In-gel fluorescence of labelled HaloTag fusion proteins

The following protocol was used to selectively label HaloTag domains exposed at the periplasmic side of the IM. Strains expressing the required Tat component-HaloTag fusions were grown to mid-exponential growth phase (OD_{600} = 0.5) in LB medium. The outer

membranes of the cells were then permeabilised using an adaptation of the method of [14]. 1 ml of cell culture was harvested by centrifugation at 10,000*g* for 2 min, washed once in phosphate buffered saline (PBS), and then resuspended in 1 ml of SET buffer (0.5 M sucrose, 0.5 mM Na_2EDTA, 100 mM Tris.HCl, pH 8.0). After incubation at 25 °C for 5 min the cells were then pelleted and resuspended in 1 ml of 1 mM $MgCl_2$ at 25 °C. The cells were then immediately pelleted by centrifugation at 4,000*g* for 2 min at 4 °C and gently resuspended in 1 ml of SET buffer. For those experiments involving PEG-Mal labelling the pellet was instead resuspended in SET buffer supplemented with 5 mM methoxypolyethylene glycol maleimide (PEG-Mal) (Sigma) and incubated for 40 min prior to further processing. The cells were then incubated for 20 min at 25 °C with 100 nM Alexa Fluor 660 HaloTag Ligand (Promega) followed by a 10 min incubation with 0.05 % (v/v) 1,3-dibromopropane (Sigma) to covalently block any unreacted Halotag [15]. In control experiments where all HaloTag proteins in the cell were to be labelled, either 1 % (w/v) dodecyl maltoside (Anatrace) was added along with the Alexa Fluor 660 Halotag ligand or the cells were treated with 10 nM cell permeable Janelia Fluor 646 HaloTag Ligand (Promega). After HaloTag labelling, the cells were centrifuged at 30,000*g* for 2 min at 4 °C and the pellet resuspended in 30 μl of SDS-loading buffer. The samples were then heated at 100 °C for 5 min, or for TatC fusions heated at 50 °C for 5 min followed by sonication (Vibra-Cell) on ice for 3 s with a 3 mm stepped microtip at 20 % amplitude. All samples were subjected to SDS-PAGE using a 4-20 % polyacrylamide gel (Biorad). In-gel fluorescence was visualised using an Amersham Typhoon 5 Biomolecular Imager equipped with a 635 nm laser and 670+/-30 bandpass filter.

Protein methods

Immunoblotting and CueO export assays were carried out as previously described [5]. The antibodies use in this study were those against TatA, TatB, TatC [16], HaloTag (Promega), and CueO [5]. Expression levels were assessed in cells grown to mid-exponential phase (OD_{600} = 0.5) in LB medium. For the analysis of TatA fusions, 1 ml of the culture was pelleted, resuspended in 50 µl of SDS-loading buffer, and heated at 100 °C for 5 min before SDS-PAGE. For the analysis of TatB and TatC fusions 250 ml of cell culture was harvested by centrifugation at 15,000*g* for 10 min at 4 °C and resuspended in 2.5 ml 50 mM Tris.HCl, 200 mM NaCl, 0.2 mg/ml lysozyme. The cells were sonicated on ice for 1 min (10 s on, 4 s off) with a 3 mm stepped microtip at 50 % amplitude and the resultant solution centrifuged at 3,500*g* for 15 min at 4 °C. The supernatant was then subject to centrifugation at 100,000*g* for 45 min at 4 °C to harvest the cell membranes. The membrane pellet was resuspended in 100 µl 50 mM Tris.HCl, 200 mM NaCl, concentrations were normalised using a DC assay (BioRad), then samples were mixed with SDS-loading buffer, heated to 50 °C for 5 min, and subjected to SDS-PAGE.

Table S1 – *E. coli* strains used in this study

Strains	Abbreviation	Genotype	Source
MC4100	ABCE	F^-, *ΔlacU169, araD139, rpsL150, relA1, ptsF, rbsR, flbB5301*	[17]
MΔBC	AE	MC4100 Δ*tatBC*	[2]
MΔABC-A	E	MC4100 (arabinose resistant) Δ*tatABC*	[2]
DADE		MC4100 Δ*tatABC* Δ*tatE*	[18]
ELV16	BCE	MC4100 Δ*tatA*	[19]
JARV16	BC	MC4100 Δ*tatA* Δ*tatE*	[19]
BW25113		F^-, *lacI*$^+$, *rrnBT14, ΔlacZWJ16, ΔaraBADAH33, ΔrhaBADLD78, rph-1, Δ(araB–D)567, Δ(rhaD–B)568, ΔlacZ4787(*::*rrnB-3), hsdR514*	[20]
BW25113 Δ*mscL*		BW25113 Δ*mscL*	[21]
SE2060		MC4100 *lamBS60*	[22]
SE6004		SE2060 *prlA4*	[23]
ELV16-A λAry	A^RyBCE	MC4100 (arabinose resistant) Δ*tatA*, *attB*::P_{tatA}*tatA-RL-eyfp*^{A206K} (kan^r)	[2]
ELV16 λA^Rd	A^RdBCE	ELV16 *attb*::P_{tatA}*tatA-RL-dL5* (kan^r)	This work
JARV16 λ$A_{F39A}{}^R$d	$A_{F39A}{}^R$dBC	JARV16 *attb*::P_{tatA}*tatA*$_{F39A}$*-RL-dL5* (kan^r)	This work
DADE λ$A_{F39A}{}^R$d	$A_{F39A}{}^R$d	DADE *attb*::P_{tatA}*tatA*$_{F39A}$*-RL-dL5* (kan^r)	This work
ELV16 λAd	AdBCE	ELV16 *attb*::P_{tatA}*tatA-dL5* (kan^r)	This work
ELV16 λA^Rh	A^RhBCE	ELV16*attb*::P_{tatA}*tatA-RL-halotag* (kan^r)	This work
ELV16 λAh	AhBCE	ELV16, *attb*::P_{tatA}*tatA-halotag* (kan^r)	This work
DADE λA^Rd	A^Rd	DADE *attb*::P_{tatA}*tatA-RL-dL5* (kan^r)	This work
MΔABC-A λA^Rd	A^RdE	MC4100 (arabinose resistant) Δ*tatABC*, *attB*::P_{tatA}*tatA-RL-dL5* (kan^r)	This work
DADE λA^Rh	A^Rh	DADE *attb*::P_{tatA}*tatA-RL-halotag* (kan^r)	This work
DADE λAh	Ah	MC4100 Δ*tatABC* Δ*tatE* *attb*::P_{tatA}*tatA-halotag* (kan^r)	This work
ELV16 λ$A_{G2C}{}^R$h	$A_{G2C}{}^R$hBCE	ELV16 *attb*::P_{tatA}*tatA*$_{G2C}$*-RL-halotag* (kan^r)	This work
ELV16 λ$A_{S35C}{}^R$h	$A_{S35C}{}^R$hBCE	ELV16 *attb*::P_{tatA}*tatA*$_{S35C}$*-RL-halotag* (kan^r)	This work
ELV16 λ$A_{E47C}{}^R$h	$A_{E47C}{}^R$hBCE	ELV16 *attb*::P_{tatA}*tatA*$_{E47C}$*-RL-halotag* (kan^r)	This work
ELV16 λ$A_{T78C}{}^R$h	$A_{T78C}{}^R$hBCE	ELV16 *attb*::P_{tatA}*tatA*$_{T78C}$*-RL-halotag* (kan^r)	This work

Table S2 – plasmids used in this study

Plasmid name	Description	Source
pQE-80	Expression vector with LacI-repressible T5 promoter, *lacI*^{q}, Amp^r.	Qiagen
pQE-80 CueO	pQE-80, synthesis of *E. coli* CueO with a C-terminal His_6 tag.	[24]
pSU18 AmiB	Synthesis of *E. coli* AmiB from a LacI-repressible *lac* promoter, *lacI*^{q}, Cam^r.	[8]
pSU40 AmiB	Synthesis of *E. coli* AmiB from a LacI-repressible *lac* promoter, *lacI*^{q}, Kan^r.	[8]
pSU18 AmiB-*lacO*	pSU18-AmiB without the Lac operator (*lacO*).	This work
pQE-80 ssTorA-dL5	pQE-80, synthesis of dL5 with a N-terminal Tat signal sequence from TorA (ssTorA).	This work
pQE-80 dL5	pQE-80, synthesis of dL5.	This work
pQE-80 HaloTag	pQE-80, synthesis of HaloTag.	This work
pTGS	*sstorA-gfpmut2.*	[6]
pQETG	pQE-80, expression of *sstorA-gfpmut2.*	This work
p101C*TatBC	Expression from the *tatA* promoter of *tatBC* with a modified RBS, Cam^r.	[2]
p101C*$TatBC_{E170A}$	p101C*TatBC, *tatC(E170A)*	[25]
p101C*TatBhC	p101C*TatBC, TatB produced with a C-terminal HaloTag	This work
p101C*TatBCh	p101C*TatBC, TatC produced with a C-terminal HaloTag	This work
p101C*TatBCd	p101C*TatBC, TatC produced with a C-terminal dL5 domain	This work
p101C*TatBdC	p101C*TatBC, TatB produced with a C-terminal dL5 domain	This work
p101C*$TatB_{F2C}hC$	p101C*TatBhC, *tatB(F2C)*	This work
p101C*$TatB_{T45C}hC$	p101C*TatBhC, *tatB(T45C)*	This work
p101C*$TatB_{P171C}hC$	p101C*TatBhC, *tatB(P171C)*	This work
p101C*$TatBC^{\Delta cys}h$	p101C*TatBCh, *tatC(C23A, C33A, C179A, C224A)*	This work
p101C*$TatBC^{\Delta cys}{}_{D40C}h$	p101C*$TatBC^{\Delta cys}h$, *tatC(D40C)*	This work
p101C*$TatBC^{\Delta cys}{}_{A65C}h$	p101C*$TatBC^{\Delta cys}h$, *tatC(A65C)*	This work
p101C*$TatBC^{\Delta cys}{}_{S148C}h$	p101C*$TatBC^{\Delta cys}h$, *tatC(S148C)*	This work
p101C*$TatBC^{\Delta cys}{}_{D211C}h$	p101C*$TatBC^{\Delta cys}h$, *tatC(D211C)*	This work
p101C*$TatBC^{\Delta cys}{}_{A251C}h$	p101C*$TatBC^{\Delta cys}h$, *tatC(A251C)*	This work

Table S3 – DNA primers used in this study

Primer name	Primer sequence	Used for construction of
dL5F	5'-ATTACTCGTACGCAGGCCGTCGTTACCCAAGAACCTAGTG-3'	pBSTatArdL5
dL5R	5'-ATGTAAGGATCCTCACTCGAGTAAGATACCGGTGGAGAGGACGG-3'	pBSTatArdL5
HaloF	5'- TTACTCGTACGGAAATCGGTACTGGCTTTCCATTCG-3'	pBSTatArhalo
HaloR	5'-TGTAAGGATCCTTAACCGGAAATCTCCAGAGTAGACAG-3'	pBSTatArhalo
NORLdL5F	5'-CAGGCCGTCGTTACCCAA-3'	pBSTatAdL5
NORLdL5R	5'-CACCTGCTCTTTATCGTGGC-3'	pBSTatAdL5
NORLhaloF	5'-CGTACGGAAATCGGTACTGG-3'	pBSTatAhalo
NORLhaloR	5'-GCCGGCATGCATCACCTG-3'	pBSTatAhalo
TATAF39AF	5'-GATCAAAGGCGCCAAAAAAGCAATGAGCG-3'	pBSTatA_{F39A}rhalo, pBSTatA_{F39A}rdL5
TATAF39AR	5'-GACGCACCAAGATCGGAA-3'	pBSTatA_{F39A}rhalo, pBSTatA_{F39A}rdL5
TATAG2CF	5'-AACATGTATGTGTGGTATCAGTATTTG-3'	pBSTatA_{G2C}rhalo
TATAG2CR	5'-CCTCTGTGGTAGATGATG-3'	pBSTatA_{G2C}rhalo
TATAS35CF	5'-TCTTGGTGCGTGTATCAAAGGCTTTAAAAAAGC-3'	pBSTatA_{S35C}rhalo
TATAS35CR	5'-TCGGAACCGATGGAGCCG-3'	pBSTatA_{S35C}rhalo
TATAE47CF	5'-GAGCGATGATTGTCCAAAGCAGG-3'	pBSTatA_{E47C}rhalo
TATAE47CR	5'-ATTGCTTTTTTAAAGCCTTTG-3'	pBSTatA_{E47C}rhalo
TATAT78CF	5'-ACAGGCTAAATGTGAAGACGCGAAGCG-3'	pBSTatA_{T78C}rhalo
TATAT78CR	5'-TCCTGATTCGTATCCGCC-3'	pBSTatA_{T78C}rhalo
TGF	5'-TAAAAGAGGAGAAATTAACTATGGACAATAACGATCTCTTTCAGGCATC-3'	pQETG
TGR	5'- TATTAGGATCCTTATTTGTATAGTTCATCCATGCCATGTGTAATCCC-3'	pQETG
TGBsiWIF	5'-GACTGACGCTCGTACGAGTAAAGGAGAAGAACTTTTCACTGGAGTTGTCCCAATT CTTGTTGAATTAGATGG-3'	pQET$_{BsiWI}$G
TGBsiWIR	5'-GCCGCTTGCGCCGCAGTC-3'	pQET$_{BsiWI}$G
BsiWIdL5F	5'-ATTACTCGTACGCAGGCCGTCGTTACCCAAGAACCTAGTG-3'	pQE-80 ssTorA-dL5
dL5BamHIR	5'-ATGTAAGGATCCTCACTCGAGTAAGATACCGGTGGAGAGGACGG-3'	pQE-80 ssTorA-dL5
delTF	5'-CAGGCCGTCGTTACCCAA-3'	pQE-80 dL5
delTR	5'- CATAGTTAATTTCTCCTCTTTAATGAATTCTGTG-3'	pQE-80 dL5
TGAgeIF	5'-GACTGACGCTACCGGTAGTAAAGGAGAAGAACTTTTCACTGGAGTTGTCCCAATTC-3'	pQET$_{AgeI}$G
TGAgeIR	5'-GCCGCTTGCGCCGCAGTC-3'	pQET$_{AgeI}$G
AgeIhaloF	5'-TTACTACCGGTGAAATCGGTACTGGCTTTCCATTCG-3'	pQE-80 HaloTag
haloBamHIR	5'-TGTAAGGATCCTTAACCGGAAATCTCCAGAGTAGACAG-3'	pQE-80 HaloTag
delTF	5'-GAAATCGGTACTGGCTTTC-3'	pQE-80 HaloTag
delThaloR	5'-CATAGTTAATTTCTCCTCTTTAATG-3'	pQE-80 HaloTag
TBHALOF	5'-CGAGTGATAAACCGCGTACGGAAATCGGTACTGGCTTTCCATTCGA-3'	p101C*TatBhC
TBHALOR	5'-GCTTAAGGTTTATCACTCGATTAACCGGAAATCTCCAGAGTAGACAGC-3'	p101C*TatBhC
TBVECTF	5'-CTCTGGAGATTTCCGGTTAATCGAGTGATAAACCTTAAGCATGTCTG-3'	p101C*TatBhC
TBVECTR	5'-GGAAAGCCAGTACCGATTTCCGTACGCGGTTTATCACTCGAC-3'	p101C*TatBhC
TBdL5F	5'- CGAGTGATAAACCGCGTACGCAGGCCGTCGTT-3'	p101C*TatBdC
TBdL5R	5'-GCTTAAGGTTTATCACTCGATCACTCGAGTAAGATACCGGTGGAG-3'	p101C*TatBdC
TBdVTF	5'-CCGGTATCTTACTCGAGTGATCGAGTGATAAACCTTAAGCATGTCTGT-3'	p101C*TatBdC
TBdVR	5'-TCTTGGGTAACGACGGCCTGCGTACGCGGTTTATCACTCGACGAAGG-3'	p101C*TatBdC
TCHALOF	5'- AAGCTTGCATGCCTGCAGCCTTAACCGGAAATCTCCAGAGT-3'	p101C*TatBCh
TCHALOR	5'- AAAGCGAAAAAACTGAAGAAGAAATCGGTACTGGCT-3'	p101C*TatBCh
TChVF	5'- GGAAAGCCAGTACCGATTTCTTCTTCAGTTTTTTCGCTTTCTGCTTCAGC-3'	p101C*TatBCh
TChVR	5'- CTCTGGAGATTTCCGGTTAAGGCTGCAGGCATGCAAG-3'	p101C*TatBCh
TCdL5F	5'-AAAGCGAAAAAACTGAAGAACAGGCCGTCGTTACCCAAG-3'	p101C*TatBCd
TCdL5R	5'-AAGCTTGCATGCCTGCAGCCTCACTCGAGTAAGATACCGGT-3'	p101C*TatBCd
TCdVTF	5'-CCGGTATCTTACTCGAGTGAGGCTGCAGGCATGCA-3'	p101C*TatBCd
TCdVR	5'-TCTTGGGTAACGACGGCCTGTTCTTCAGTTTTTTCGCTTTCTGCTTCAGC-3'	p101C*TatBCd
TatCF	5'-TCGAGTGATAAACCTTAAGCATGTCTGTAGAAGATACTCAACCGCT-3'	p101C*TatB$C^{\Delta cys}$h (D40C, A65C, S148C, D211C)
TatCR	5'-CCAGTACCGATTTCCGTACGTTCTTCAGTTTTTTCGCTTTCTGCTTCAGC-3'	p101C*TatB$C^{\Delta cys}$h (D40C, A65C, S148C, D211C)
TCVF	5'-AAAGCGAAAAAACTGAAGAACGTACGGAAATC-3'	p101C*TatB$C^{\Delta cys}$h (D40C, A65C, S148C, D211C)
TCVR	5'-TGAGTATCTTCTACAGACATGCTTAAGGTTTATCAC-3'	p101C*TatB$C^{\Delta cys}$h (D40C, A65C, S148C, D211C)
TC251R	5'-CCAGTACCGATTTCCGTACGTTCTTCAGTTTTTTCGCTTTCACATTCAGCG-3'	p101C*TatB$C^{\Delta cys}$h A251C
TATBF2CF	5'-GGATCCGTGTGTGATATCGGT-3'	p101C*TatB_{F2C}hC

TATBF2CR	5′-TCCTCTGTGGTAGATGATG-3′	p101C*TatB$_{F2C}$hC
TATBT45CF	5′-ACTGGCGACATGTGTGCAGAACGAACTGACC-3′	p101C*TatB$_{T45C}$hC
TATBT45CR	5′- GAACGCAACGCGCGAATC-3′	p101C*TatB$_{T45C}$hC
TATBP171CF	5′-GAGTGATAAATGTCGTACGGAAATCGGTACTGGC-3′	p101C*TatB$_{P171C}$hC
TATBP171CR	5′- GACGAAGGGGAAGGTGCA-3′	p101C*TatB$_{P171C}$hC
D_lacO_F	5′-TTCCACACAACATACGAG-3′	pSU18 AmiB-*lacO*
D_lacO_R	5′-TTTCACACAGGAAACAGC-3′	pSU18 AmiB-*lacO*

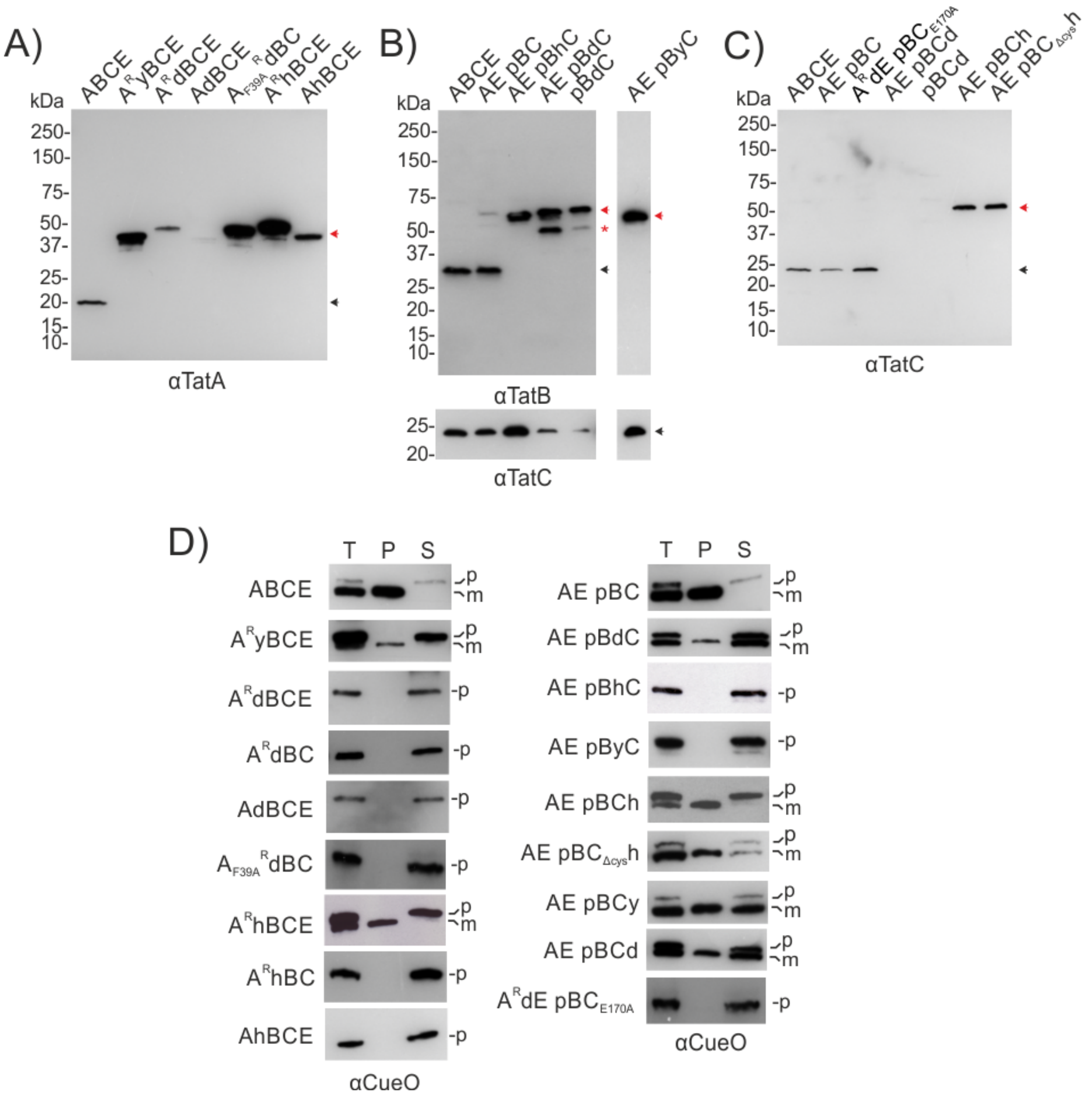

Figure S1 - Expression levels and Tat activity of dL5 and HaloTag fusions to Tat components. (**A-C**) The expression levels of (**A**) TatA, (**B**) TatB, and (**C**) TatC fusion proteins in the indicated strains were quantified by immunoblotting whole cells extracts (TatA) or purified membranes (TatB and TatC) with TatA, TatB and TatC antibodies. The black arrow indicates the immunoreactive band corresponding to the native protein and the red arrow the band corresponding to the fusion protein. * indicates a truncated form of TatB arising from partial proteolysis during membrane isolation. (**D**) The effects of reporter fusions to Tat components on the export of the Tat substrate CueO. Total cell (T), periplasm (P), and spheroplast (S) fractions of the indicated strains overproducing CueO from plasmid pQE-80 CueO were immunoblotted with antibodies against CueO. p and m designate pre-CueO and mature-CueO. Strains are named for the Tat proteins they produce with amino acid substitutions given as subscripts to the protein in which they are located (with \`$C_{\Delta cys}$' indicating a TatC variant in which all Cys residues have been replaced with Ala residues), \`p' indicating that the proteins that follow are expressed at native levels from a plasmid, and \`d', \`y' and \`h' indicating fusion to the N-terminus of dL5, YFP, and HaloTag respectively with a superscript \`R' indicating that the fusion proteins are linked by a rigid linker sequence.

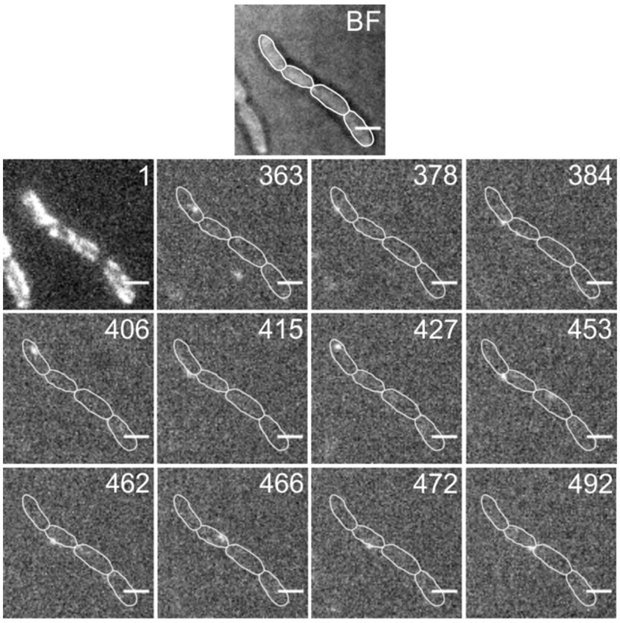

Figure S2 – Leakage of dL5 to the periplasm still occurs at elevated levels of dL5 expression - supporting data for Figure 3. Cytoplasmic dL5 was expressed from plasmid pQE-80 dL5 under inducing conditions in the *tat* mutant DADE. The cells were labelled with se-red-xc dye and imaged by stroboscopy on a 33.3 ms on to 99.9 ms off laser duty cycle. Shown are a bright field image of the cells (BF) and fluorescence images at the indicated frame numbers. Multiple foci are present at frame 1. The other frames show the trajectory of the last molecule to photobleach demonstrating movement between cells in the chain. Scale bars = 2 µm.

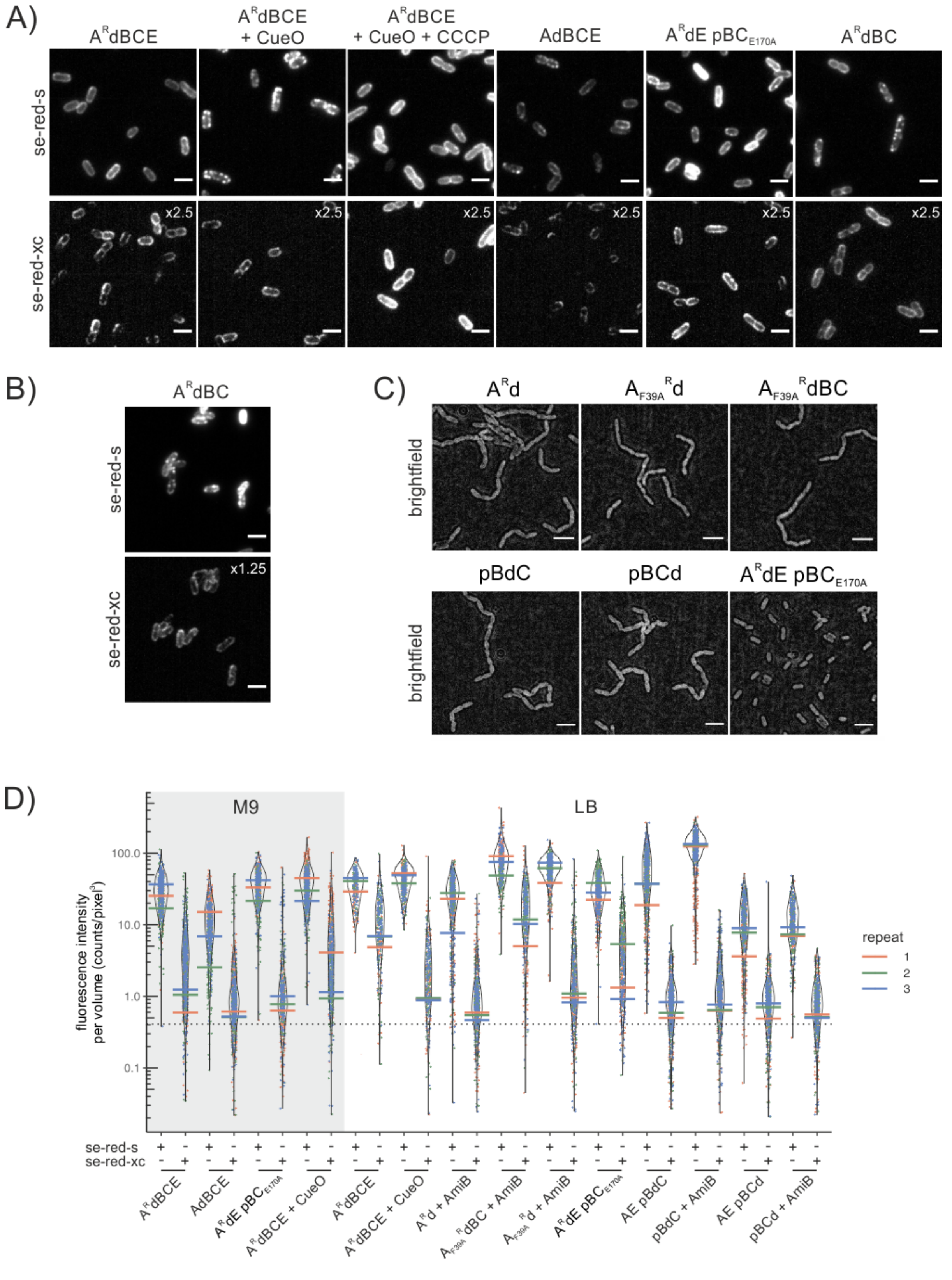

Figure S3 – Periplasmic exposure of dL5 fused to the C-terminus of TatA, TatB, or TatC - supporting data for Figure 4. (**A-B**) Fluorescence images of the indicated strains grown in (A) M9 minimal medium or (**B**) LB medium in the presence of either the cell-permeable se-red-s dye or the cytoplasm-excluded se-red-xc dye. The images correspond to the maximum intensity Z-projection of 250 frames. The brightness is standardised across the fluorescence

images in each panel. The multiplication signs indicate where the brightness has been increased. Strains are named for the Tat proteins they produce with amino acid substitutions given as superscripts to the protein in which they are located, `p' indicating that the proteins that follow are expressed at native levels from a plasmid, and `d' and `^{R}d' indicating fusion to the N-terminus of dL5 with or without a rigid linker sequence. For + CueO cells, the Tat substrate CueO was overproduced from plasmid pQE-80 CueO by induction with 1 mM IPTG for 20 mins. For + CCCP images, the cells were treated with 50 μM of the protonophore CCCP for 1 min. Dyes were added to the cells following these procedures. The image data are representative of cells from at least three independent cultures. Scale bars = 3 µm. (**C**) Brightfield images showing the cell chaining phenotypes of strains grown in LB medium without AmiB overproduction. Scale bars = 3 µm. (**D**) Distribution plots of the fluorescence intensity per cell volume from three biological repeats for each experimental condition for >100 individual cells per repeat. For pAmiB strains, AmiB was overproduced from plasmid pSU18 AmiB (A^{R}d, $A_{F39A}{}^{R}$dBC and $A_{F39A}{}^{R}$d) or pSU40 AmiB (pBdC and pBCd) to suppress the cell chaining phenotype associated with loss of Tat function. The strains were cultured in either M9 medium (grey background) or LB medium (white background). The cells were imaged under identical acquisition settings. Each colour represents a biological repeat, each point represents a single cell measurement, and the coloured horizontal bars represent the peak intensity of each biological repeat. The density of each full data set is shown by the violin plot. The horizontal dotted line represents the mean intensity per volume of cells imaged with no dye present (background autofluorescence).

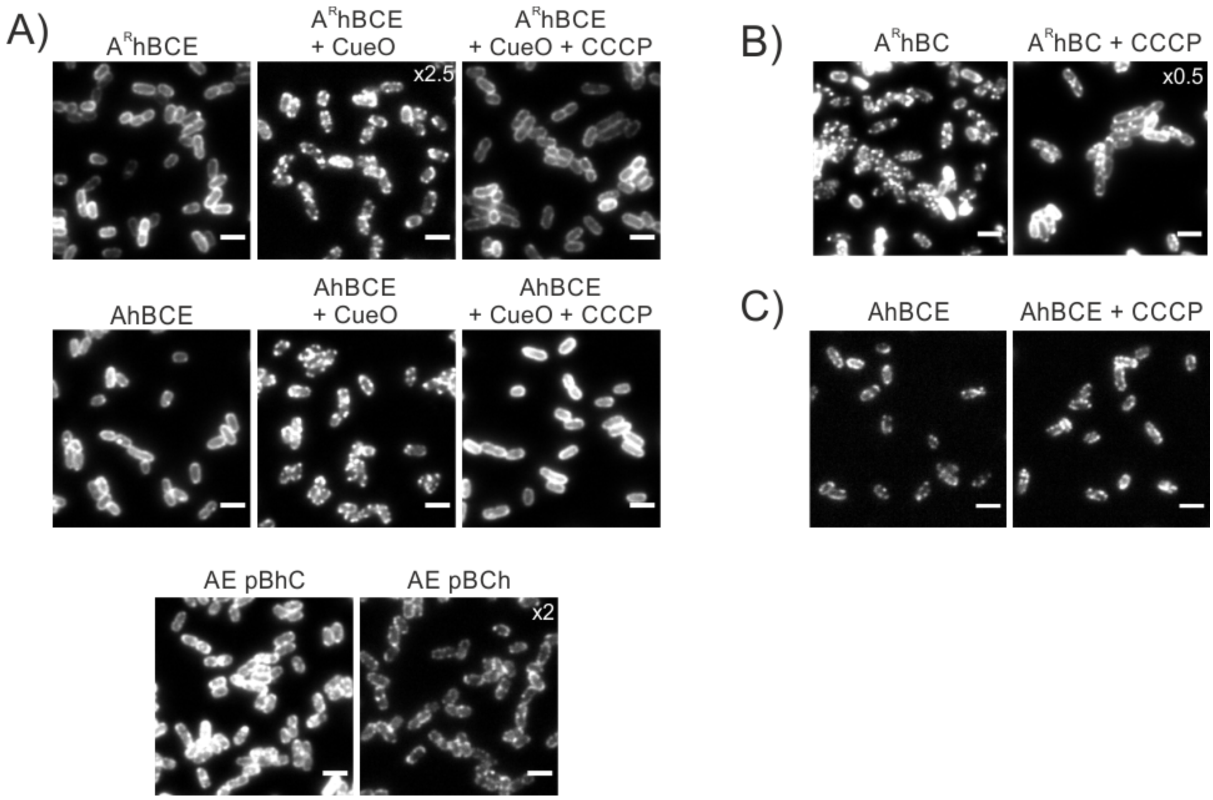

Figure S4 – Periplasmic exposure of HaloTag fused to the C-terminus of TatA, TatB, or TatC - supporting data for Figure 5. (**A-C**) Single frame (33.3 ms exposure) fluorescence images of the indicated strains grown in (**A-B**) M9 medium or (**C**) LB medium, then labelled with cell permeable Janelia Fluor-646-HaloTag ligand. In + CueO cells the Tat substrate CueO was overproduced from plasmid pQE-80 CueO by induction with 1 mM IPTG for 20 min before dye labelling. + CCCP indicates that after labelling and washing, the cells were imaged in the presence of 50µM of the protonophore CCCP. The brightness is standardised across all images. The multiplication sign indicates where the brightness has been increased. The image data are representative of cells from at least three independent cultures. Scale bars = 3 µm. Strains are named for the Tat proteins they produce with `p' indicating that the proteins that follow are expressed at native levels from a plasmid, and `h' and `^{R}h' indicating fusion to the N-terminus of HaloTag with or without a rigid linker sequence.

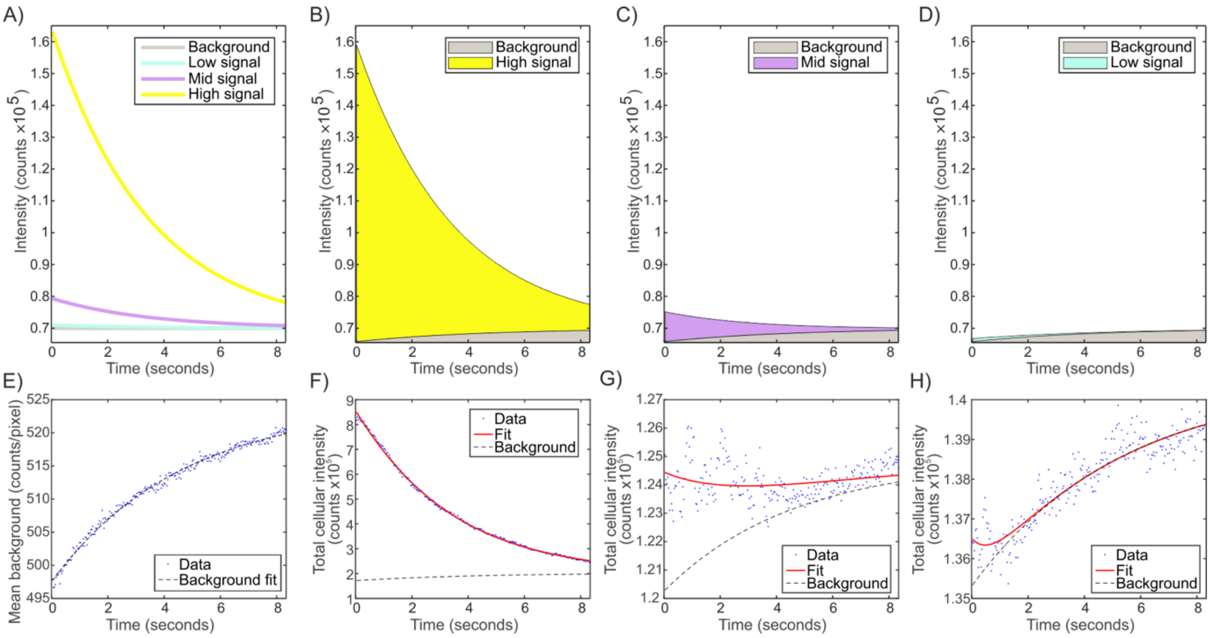

Figure S5 – Correction of fluorescence signal intensity for variation in illumination power. (**A**) Simulated High (yellow), Mid (lilac) and Low (cyan) fluorescence signals for illumination at constant power. The fluorescence signal decreases as fluorophores stochastically photobleach. (**B**-**D**) Simulated contributions of the increase in background autofluorescence (grey) and fluorescence signal (colours; same High (yellow), Mid (lilac) and Low (cyan) signals as in panel A) when illumination power increases over time to the constant level used in panel A. The simulations demonstrate that the increase in background autofluorescence has a substantial effect on the measured fluorescence of low fluorescence samples. (**E**) Example fit (black dashed line) to experimental mean background over time data (blue points) used to determine the increase in illumination power over time. (**F**-**H**) Example fits (red line) to cellular fluorescence intensity data from this study (blue points) taking into account the increasing background autofluorescence level (black dashed line) due to varying illumination power. (**F**) high, (**G**) mid and (**H**) low cellular fluorescence examples are shown.

Video S1. Trajectory of a dL5 molecule labelled with se-red-xc dye in chained cells of a Δ*tat* mutant, corresponding to Fig. 2F. The fluorescence was imaged by stroboscopy (33.3 ms on to 99.9 ms off laser duty cycle). The playback speed is 20 frames per second corresponding to 2.7x actual speed. The fluorescence video is overlaid with cell outlines (red). Scale bar = 2 µm.

[1] Bertani G. Studies on lysogenesis. I. The mode of phage liberation by lysogenic *Escherichia coli*. J Bacteriol. 1951;62:293-300.

[2] Alcock F, Baker MA, Greene NP, Palmer T, Wallace MI, Berks BC. Live cell imaging shows reversible assembly of the TatA component of the twin-arginine protein transport system. Proc Natl Acad Sci U S A. 2013;110:E3650–E9.

[3] Wang Y, Telmer CA, Schmidt BF, Franke JD, Ort S, Arndt-Jovin DJ, et al. Fluorogen activating protein-affibody probes: modular, no-wash measurement of epidermal growth factor receptors. Bioconjug Chem. 2015;26:137-44.

[4] Simons RW, Houman F, Kleckner N. Improved single and multicopy *lac*-based cloning vectors for protein and operon fusions. Gene. 1987;53:85-96.

[5] Alcock F, Damen MP, Levring J, Berks BC. *In vivo* experiments do not support the charge zipper model for Tat translocase assembly. Elife. 2017;6.

[6] DeLisa MP, Samuelson P, Palmer T, Georgiou G. Genetic analysis of the twin arginine translocator secretion pathway in bacteria. J Biol Chem. 2002;277:29825-31.

[7] Cleon F, Habersetzer J, Alcock F, Kneuper H, Stansfeld PJ, Basit H, et al. The TatC component of the twin-arginine protein translocase functions as an obligate oligomer. Mol Microbiol. 2015.

[8] Ize B, Stanley NR, Buchanan G, Palmer T. Role of the *Escherichia coli* Tat pathway in outer membrane integrity. Mol Microbiol. 2003;48:1183-93.

[9] Sambrook J, Russell DW. Molecular cloning: a laboratory manual. 3rd ed. New York: Cold Spring Harbour Press; 2001.

[10] Holleran J, Brown D, Fuhrman MH, Adler SA, Fisher GW, Jarvik JW. Fluorogen-activating proteins as biosensors of cell-surface proteins in living cells. Cytometry A. 2010;77:776-82.

[11] Schindelin J, Arganda-Carreras I, Frise E, Kaynig V, Longair M, Pietzsch T, et al. Fiji: an open-source platform for biological-image analysis. Nat Methods. 2012;9:676-82.

[12] Vincent L, Soille P. Watersheds in Digital Spaces - an Efficient Algorithm Based on Immersion Simulations. Ieee Transactions on Pattern Analysis and Machine Intelligence. 1991;13:583-98.

[13] Ducret A, Quardokus EM, Brun YV. MicrobeJ, a tool for high throughput bacterial cell detection and quantitative analysis. Nat Microbiol. 2016;1:16077.

[14] Malherbe G, Humphreys DP, Dave E. A robust fractionation method for protein subcellular localization studies in *Escherichia coli*. Biotechniques. 2019;66:171-8.

[15] Bosma T, Pikkemaat MG, Kingma J, Dijk J, Janssen DB. Steady-state and pre-steady-state kinetic analysis of halopropane conversion by a *Rhodococcus* haloalkane dehalogenase. Biochemistry. 2003;42:8047-53.

[16] Alcock F, Stansfeld PJ, Basit H, Habersetzer J, Baker MA, Palmer T, et al. Assembling the Tat protein translocase. Elife. 2016;5.

[17] Casadaban MJ, Cohen SN. Lactose genes fused to exogenous promoters in one step using a *Mu-lac* bacteriophage: *in vivo* probe for transcriptional control sequences. Proc Natl Acad Sci U S A. 1979;76:4530-3.

[18] Wexler M, Sargent F, Jack RL, Stanley NR, Bogsch EG, Robinson C, et al. TatD is a cytoplasmic protein with DNase activity. No requirement for TatD family proteins in sec-independent protein export. J Biol Chem. 2000;275:16717-22.

[19] Sargent F, Stanley NR, Berks BC, Palmer T. Sec-independent protein translocation in *Escherichia coli*. A distinct and pivotal role for the TatB protein. J Biol Chem. 1999;274:36073-82.

[20] Datsenko KA, Wanner BL. One-step inactivation of chromosomal genes in *Escherichia coli* K-12 using PCR products. Proc Natl Acad Sci U S A. 2000;97:6640-5.

[21] Baba T, Ara T, Hasegawa M, Takai Y, Okumura Y, Baba M, et al. Construction of *Escherichia coli* K-12 in-frame, single-gene knockout mutants: the Keio collection. Molecular systems biology. 2006;2:2006 0008.

[22] Emr SD, Silhavy TJ. Mutations Affecting localization of an *Escherichia coli* outer membrane protein, the bacteriophage lambda receptor. Journal of Molecular Biology. 1980;141:63-90.

[23] Emr SD, Hanley-Way S, Silhavy TJ. Suppressor mutations that restore export of a protein with a defective signal sequence. Cell. 1981;23:79-88.

[24] Leake MC, Greene NP, Godun RM, Granjon T, Buchanan G, Chen S, et al. Variable stoichiometry of the TatA component of the twin-arginine protein transport system observed by *in vivo* single-molecule imaging. Proc Natl Acad Sci U S A. 2008;105:15376-81.

[25] Alcock F, Berks BC. New insights into the Tat protein transport cycle from characterizing the assembled Tat translocon. Mol Microbiol. 2022;118:637-51.
